# The contribution of historical processes to contemporary extinction risk in placental mammals

**DOI:** 10.1126/science.abn5856

**Published:** 2023-04-28

**Authors:** Aryn P. Wilder, Megan A Supple, Ayshwarya Subramanian, Anish Mudide, Ross Swofford, Aitor Serres-Armero, Cynthia Steiner, Klaus-Peter Koepfli, Diane P. Genereux, Elinor K. Karlsson, Kerstin Lindblad-Toh, Tomas Marques-Bonet, Violeta Munoz Fuentes, Kathleen Foley, Wynn K. Meyer, Zoonomia Consortium, Oliver A. Ryder, Beth Shapiro

**Affiliations:** 1 Conservation Genetics, San Diego Zoo Wildlife Alliance; Escondido, CA 92027, USA.; 2 Department of Ecology and Evolutionary Biology, University of California Santa Cruz; Santa Cruz, CA 95064, USA.; 3 Howard Hughes Medical Institute, University of California Santa Cruz; Santa Cruz, CA 95064, USA.; 4 Broad Institute of MIT and Harvard; Cambridge, MA 02139, USA.; 5 Phillips Exeter Academy; Exeter, NH 03833, USA.; 6 Institute of Evolutionary Biology, Department of Experimental and Health Sciences, Universitat Pompeu Fabra; Barcelona, 08003, Spain.; 7 Smithsonian-Mason School of Conservation, George Mason University; Front Royal, VA 22630, USA.; 8 Center for Species Survival, Smithsonian Conservation Biology Institute, National Zoological Park; Washington, DC, 30008, USA.; 9 Computer Technologies Laboratory, ITMO University; St. Petersburg, 197101, Russia.; 10 Program in Bioinformatics and Integrative Biology, University of Massachusetts Medical School; Worcester, MA 01605, USA.; 11 Science for Life Laboratory, Department of Medical Biochemistry and Microbiology, Uppsala University; Uppsala, 751 32, Sweden.; 12 Catalan Institution of Research and Advanced Studies; Barcelona, 08010, Spain.; 13 Centre for Genomic Regulation, Barcelona Institute of Science and Technology; Barcelona, 08028, Spain.; 14 Institut Català de Paleontologia Miquel Crusafont, Universitat Autònoma de Barcelona; Barcelona, 08193, Spain.; 15 European Molecular Biology Laboratory-European Bioinformatics Institute, Wellcome Genome Campus; Hinxton, UK.; 16 College of Law, University of Iowa; Iowa City, IA 52242, USA.; 17 Lehigh University, Biological Sciences; Bethlehem, PA 18015, USA.; 18 Department of Evolution, Behavior and Ecology, Division of Biology, University of California, San Diego; La Jolla, CA 92039 USA.

## Abstract

Species persistence can be influenced by the amount, type, and distribution of diversity across the genome, suggesting a potential relationship between historical demography and resilience. Here, we surveyed genetic variation across single genomes of 240 mammals comprising the Zoonomia alignment to evaluate how historical effective population size (*N*_*e*_) impacts heterozygosity and deleterious genetic load and how these factors may contribute to extinction risk. We find that species with smaller historical *N*_*e*_ carry a proportionally larger burden of deleterious alleles due to long-term accumulation and fixation of genetic load, and have higher risk of extinction. This suggests that historical demography can inform contemporary resilience. Models that included genomic data were predictive of species’ conservation status, suggesting that, in the absence of adequate census or ecological data, genomic information may provide an initial risk assessment.

## One-Sentence Summary

Genomic data from 240 species show that information encoded within a single genome can provide a conservation risk assessment.

The current rate of biodiversity loss amounts to a sixth mass extinction(1) and is compounded by substantial population declines across nearly one third of vertebrate species(2). Many species need immediate conservation intervention, a process that is especially challenging for the more than 20,000 species currently listed as “Data Deficient” by the International Union for Conservation of Nature (IUCN). Fortunately, genomic data, which are increasingly available for a broad taxonomic range of species, may hold promise for helping to identify at-risk species by providing readily accessible information on demography and fitness-relevant genetic variation(3, 4). It remains poorly explored, however, to what extent genomic data on their own are sufficient to help triage endangered species for conservation intervention.

Population genetic diversity and individual heterozygosity are long recognized correlates of fitness-relevant functional variation(5, 6). Our previous analysis of 124 placental mammalian genomes showed that lower heterozygosity and stretches of homozygosity are more common in species in threatened IUCN Red List categories(7). However, functional diversity, including estimates of adaptive variation and genetic load, may also be useful correlates of population resiliency. Such measures are increasingly accessible with emerging genomic tools(8) and comparative genomics resources such as the Zoonomia alignment of placental mammalian genomes (table S1)(7). The Zoonomia alignment provides high-resolution constraint scores and reconstructed ancestral sequences that can help to identify deleterious alleles at functionally important sites(7, 9).

Here, we surveyed the distribution of neutral and functional genetic variation across 240 species in the Zoonomia alignment to determine how historical effective population sizes (*N*_*e*_) have influenced heterozygosity and deleterious genetic load (fig. S1). We test the value of genomic data to more precisely target species for conservation efforts by comparing the outcome of predictive models of conservation status that use ecological data, genomic data, or both. While we acknowledge the limitations of assuming that single genomes are representative of a species, our approach capitalizes on the unique resource provided by the Zoonomia consortium to explore whether genomic data can provide initial risk assessments that may be useful to triage data-deficient species and guide resource allocation for conservation intervention.

## Historical population size is relevant to contemporary extinction risk

**Fig. 1.**
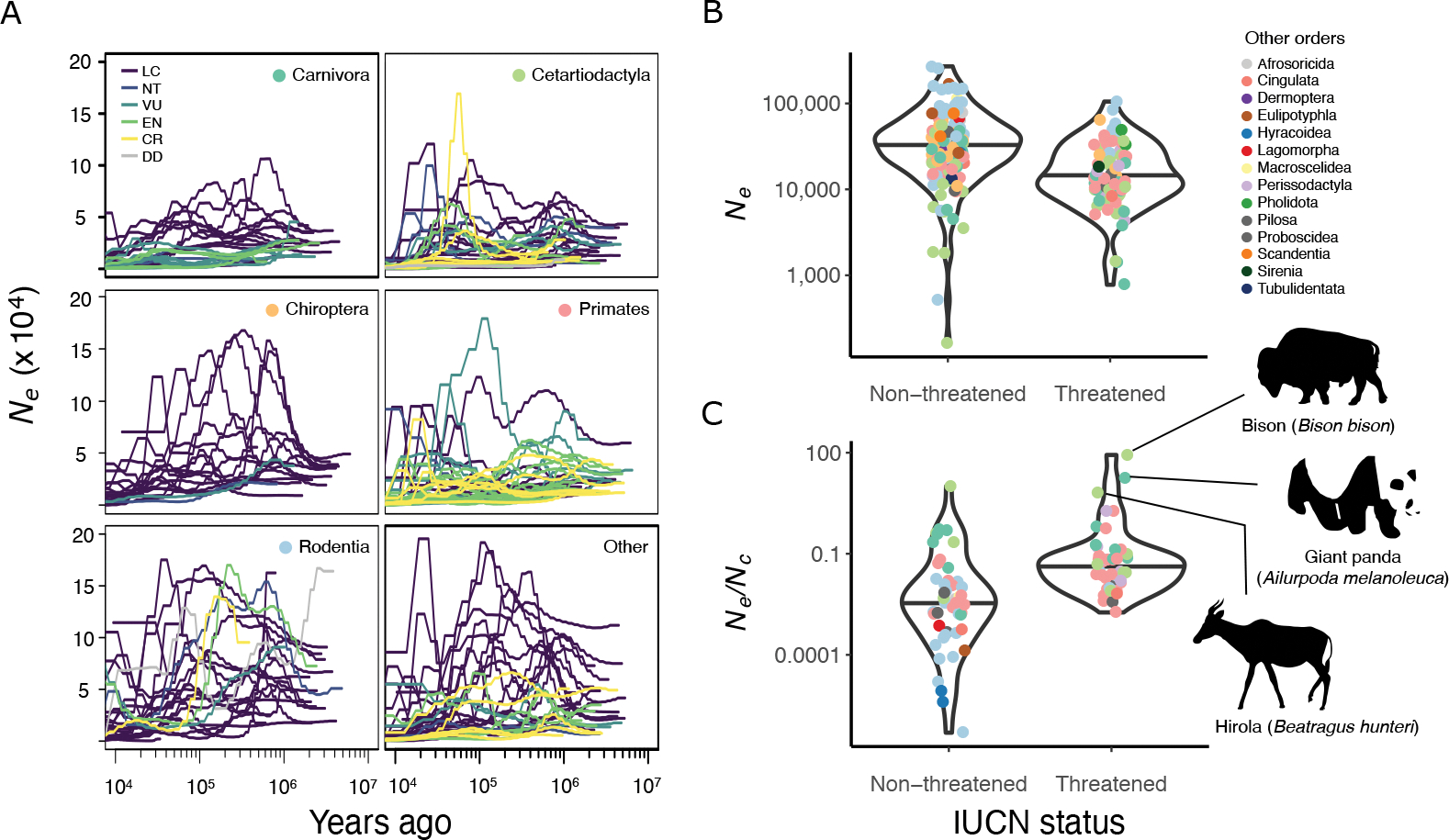
Demographic history across mammalian orders and IUCN Red List categories. **(A)** Estimates of effective population sizes (*N*_*e*_) over time displayed by taxonomic order. Lines represent individual species, colored by IUCN status (LC= Least Concern, NT=Near Threatened, VU=Vulnerable, EN=Endangered, CR=Critically Endangered, DD=Data Deficient). Colored dots correspond to the taxonomic order of species depicted in **(B)** and **(C)**. For visualization, only species with *N*_*e*_ estimates under 200,000 for every time point are shown. **(B)** Harmonic mean *N*_*e*_ was significantly lower in threatened IUCN categories relative to non-threatened (phylolm, p<3.3e-5). **(C)** The ratio of historical *N*_*e*_ to contemporary census population size (*N*_*e*_/*N*_*c*_) can identify species with smaller *N*_*c*_ than expected from historical *N*_*e*_ (phylolm, p=0.012). Points in **(B)** and **(C)** show individual species, colored by taxonomic order.

Species with historically small *N*_*e*_ tend to be classified in threatened IUCN Red List categories (Fig. 1). Species classified as Near Threatened (NT), Vulnerable (VU), Endangered (EN) or Critically Endangered (CR) had significantly smaller harmonic mean *N*_*e*_ (mean_threatened_=18,950) compared to non-threatened species (Least Concern (LC); mean_non-threatened_=27,839; p<3.3e-5 when accounting for relationships across the phylogeny; Fig. 1B; figs. S2). *N*_*e*_ was also significantly smaller in threatened compared to non-threatened species within two of three taxonomic orders with sufficient numbers of species to test (Cetartiodactyla: mean_threatened_=18,336, mean_non-threatened_=22,648, p=0.023; and Carnivora: mean_threatened_=9,636, mean_non-threatened_=26,195, p=2.4e-5; but not Primates: mean_threatened_=22,508, mean_nonthreatened_=24,373, p=0.31; fig. S3). Within these two orders in particular, large-bodied herbivores and carnivores have declined in both geographic range and population size during the Anthropocene(10, 11). Smaller populations are expected to have higher extinction risk, yet these historical *N*_*e*_ estimates reflect periods more than 10,000 years in the past, suggesting that long-term characteristics of ancestral populations can be informative about population size and extinction risk today. These results support the utility of metrics of genome-wide diversity in conservation assessments, a topic that is currently debated(12, 13).

Estimates of historical *N*_*e*_ can also identify previously large populations that have experienced contemporary declines. Specifically, if the estimate of historical *N*_*e*_ is large while *N*_*c*_ is small, this inflates the *N*_*e*_/*N*_*c*_ ratio. In a study of pinnipeds, for example, most species that had undergone recent declines had smaller population census sizes (*N*_*c*_) than expected based on their historical *N*_*e*_ *(14)*. To test this across the taxonomic range of the Zoonomia alignment, we examined the ratio of deep historical *N*_*e*_ to contemporary *N*_*c*_ for 89 species with population census information available in PanTHERIA(15). Species in threatened IUCN categories had larger *N*_*e*_/*N*_*c*_ ratios, i.e. smaller contemporary *N*_*c*_ relative to historical *N*_*e*_ (mean_threatened_=1.07e-3; mean_non-threatened_=4.29e-4; p=0.012; Fig. 1C). The relationship was also significant within Primates (phylolm, mean_threatened_=3.46e-3; mean_non-threatened_=1.11e-3; p=0.029), the only order with available *N*_*e*_/*N*_*c*_ estimates and sufficient numbers of taxa in the two threat categories, indicating that the pattern holds among species with similar life-history traits. Across taxa, the largest *N*_*e*_/*N*_*c*_ ratios included American bison (*Bison bison*), giant panda (*Ailuropoda melanoleuca*), and hirola (*Beatragus hunteri*), all of which have declined due to recent human activities(16–18).

## Historically smaller populations carry proportionally larger burdens of genetic load

Historical *N*_*e*_ is correlated with the proportion of deleterious substitutions in mammalian genomes, reflecting the accumulation and fixation of genetic load over long evolutionary time periods. We called derived, single nucleotide substitutions for each species relative to the reconstructed sequence of the nearest ancestral phylogenetic node and called heterozygous sites from resequencing data mapped to the focal genome. We inferred the impacts of derived substitutions and heterozygous variants assuming that mutations at sites that are conserved across taxa (phyloP>2.27)(9) and nonsynonymous mutations are predominantly deleterious (fig. S1)(19). Assuming most substitutions are fixed and mutation rates are similar across the phylogeny (20)(21), the proportion of substitutions that are deleterious should be correlated with the total number of fixed deleterious mutations in the genome. Deleterious substitutions should therefore largely reflect fixed drift load that reduces the mean fitness of the population, whereas heterozygous deleterious variants reflect segregating mutational load(22).

**Fig. 2.**
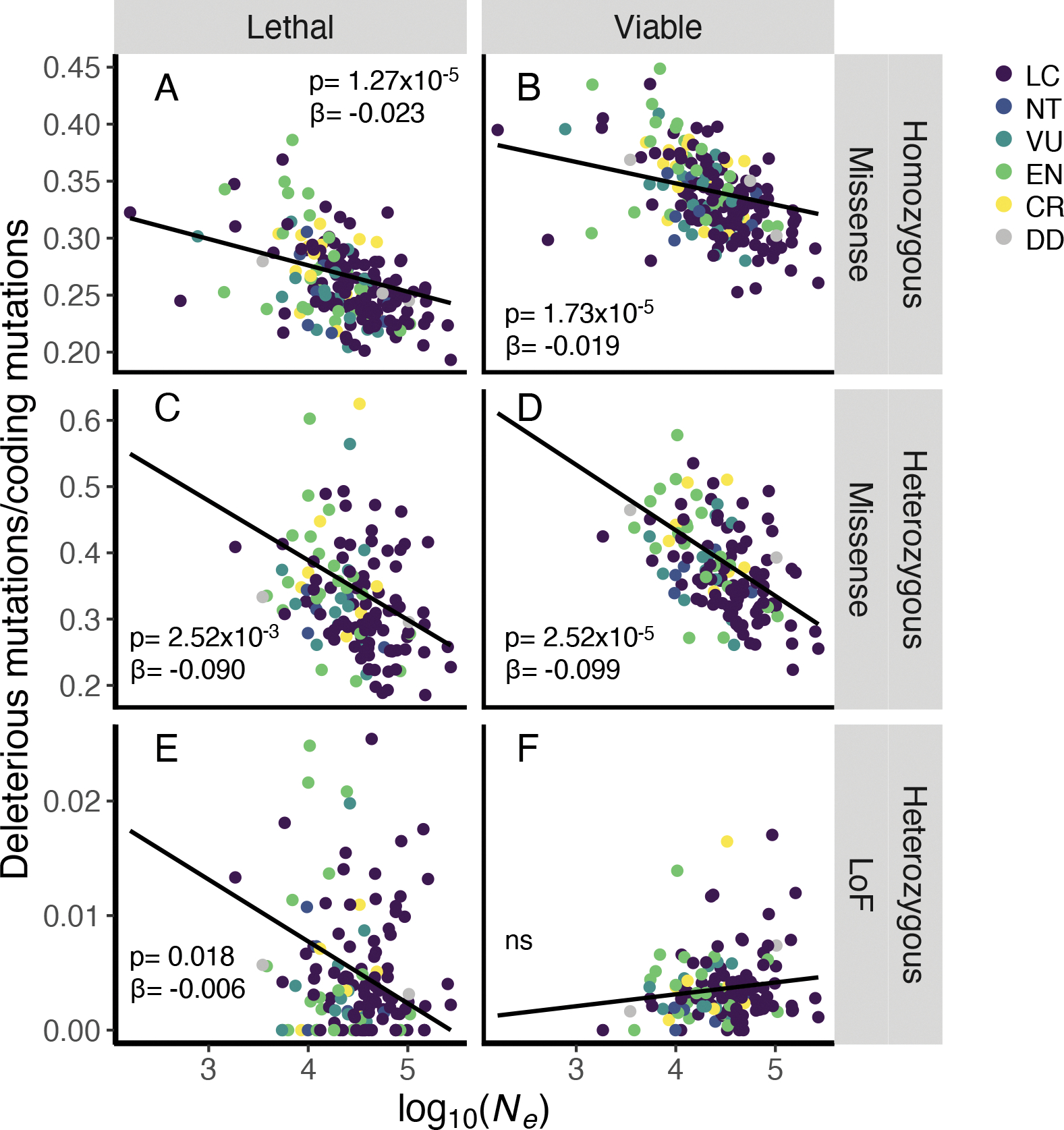
Historically small populations have more deleterious genetic load in protein-coding genes. Proportion of homozygous missense substitutions **(A-B)**, heterozygous missense variants **(C-D)** and heterozygous loss-of-function variants **(E-F)** in genes as a function of historical *N*_*e*_ across species. Genes were classified by associated lethal or viable phenotypes in knockout mice. Proportions of heterozygous and homozygous missense mutations were negatively correlated with *N*_*e*_ (all p<0.052), whereas heterozygous loss-of-function alleles were not consistently correlated with *N*_*e*_. Phylogenetically corrected p-values and coefficients (phylolm) are reported.

We found that species with smaller *N*_*e*_ had proportionally more substitutions at evolutionarily conserved sites genome-wide (phylolm, p=9.65e-3) and proportionally more missense substitutions in genes (phylolm, p=7.76e-5; fig. S4). Phylop kurtosis, which describes the extreme phyloP outliers in the tail of the distribution across substitutions, was positively correlated with *N*_*e*_ (phylolm, p=0.014). This means that species with smaller *N*_*e*_ had smaller right tails and therefore fewer substitutions at extremely conserved sites. To further parse potential fitness impacts of mutations in protein-coding regions, we examined genes with associated viability phenotypes in single-gene knockout mouse lines classified by the International Mouse Phenotyping Consortium (IMPC), assuming that, when aggregated across many genes, viability classifications are correlated to their fitness impacts in other species(23). Species with smaller *N*_*e*_ had proportionally more missense mutations relative to coding mutations in nearly all categories (phylolm, p<3.00e-5; Fig. 2; figs. S5–S6). We observed proportionally fewer missense mutations in IMPC lethal genes relative to IMPC viable genes (ANOVA, p<4.42e-9; fig. S7), reflecting stronger purifying selection in the lethal gene class, but the negative correlation was nonetheless consistent for both lethal and viable categories (Fig. 2). This relationship supports both theoretical predictions that smaller populations experiencing strong drift accumulate and fix weakly and moderately deleterious alleles (drift load)(12, 24) and empirical studies involving fewer or single taxa(25–27).

The correlations between *N*_*e*_ and conservation status and between *N*_*e*_ and drift load suggests that historical demography may influence contemporary extinction risk by shaping genome-wide diversity and genetic load. We found inconsistent relationships, however, between a species’ proportional genetic load and its odds of being threatened. Species with proportionally more missense substitutions were more likely to be threatened when considering all genes (phyloglm, p=0.002; fig. S4D), as well as genes in lethal and viable IMPC categories (phyloglm, p<0.023; fig. S6), as observed in other taxa(28). Drift load estimated from evolutionary constraint across the genome, however, showed the opposite pattern: species with proportionally fewer substitutions at evolutionarily conserved sites were more likely to be threatened (phyloglm, p=1.38e-05; fig. S4C). This latter result contrasts with expectations, given that threatened species have smaller *N*_*e*_ on average (Fig 1) and smaller *N*_*e*_ is associated with proportionally more substitutions at conserved sites (phylolm, p=9.6e-3; fig. S4A). Interestingly, a previous study of 100 mammal genomes also found that threatened species had lower mean conservation scores across mutations(29). They suggested that the pattern may reflect fewer recessive deleterious alleles due to purging or the loss of these rare alleles to drift. The conflicting relationships between conservation status and metrics of drift load thus do not provide strong support for a mechanistic link between fixed drift load as measured in this study and species’ resilience against extinction.

## Genomic information can help predict extinction risk

**Fig. 3.**
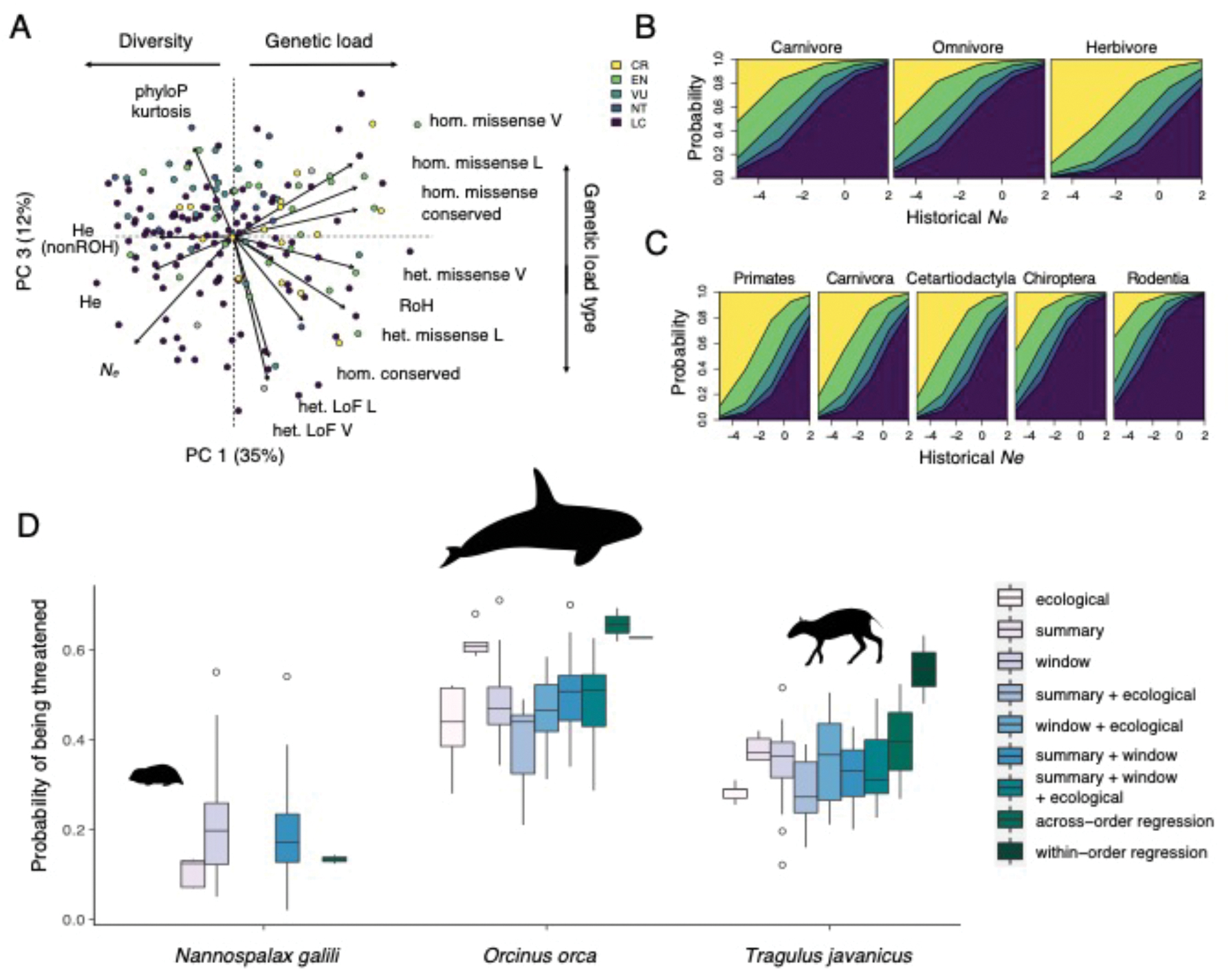
Prediction of conservation status of species using genomic information. **(A)** Principal components (PCs) that significantly predict threatened status. PC1 describes heterozygosity, *N*_*e*_ and deleterious variation, and PC3 distinguishes types of deleterious variation. Loadings of genomic variables (arrows; table S3) are labeled as described in table S2 (L=IMPC lethal genes; V=IMPC viable genes). Points indicate species, colored by IUCN status as shown in (B). **(B-C)** Probability of assignment to IUCN categories by diet and scaled values of historical *N*_*e*_ **(B)**, and by taxonomic order and historical *N*_*e*_ of species **(C)**. Decreased historical *N*_*e*_ is consistently associated with increased risk, but the magnitude varies by diet and taxonomic order. **(D)** Conservation status predictions for three data deficient species using random forest models with window-based metrics (windows), ecological variables (ecological), and/or genome-wide summary variables (summary), and predictions from regression models within and across taxonomic orders. *Nannospalax galili* lacked ecological data and adequate within-order data, so only predictions from across-order regression and windows models are shown for this species.

Historical *N*_*e*_ was the most consistent genomic predictor of conservation status across regression models, while the predictive value of genetic load metrics varied with phylogenetic context (Fig. 3, tables S2–S3). Ordinal and logistic regression models incorporating genomic variables with taxonomic order and dietary trophic level showed that the effect of *N*_*e*_ varied by ecological context. For example, an herbivore with a given *N*_*e*_ was more likely to be threatened than a carnivore or omnivore with the same *N*_*e*_ (Fig. 3B), supporting findings of elevated extinction risk in herbivores despite larger populations(30). Similarly, Carnivora and Primates both had increased risk with lower levels of severely deleterious genetic load. However, the specific metric of load that predicted conservation status differed among taxonomic orders, perhaps reflecting differences in natural history or ecological flexibility (figs. S8–S10). Principal components (PC) regression of demographic and genetic load variables showed that, overall, threatened species tended to have proportionally more deleterious mutations in coding regions, lower heterozygosity, and smaller *N*_*e*_ (PC1; p=0.0038), as well as proportionally more missense substitutions (PC3; p=5.6e-4; Fig. 3A, table S3). Although no single genomic variable unambiguously discriminated threatened from non-threatened species (fig. S2), many have predictive value, which will be particularly relevant for species lacking adequate ecological or census data.

Although ecological data were more powerful than genomic data to predict extinction risk in our predictive models, models using only information from single genomes nonetheless identified species at risk of being threatened. We generated random forest models to predict conservation status from ecological traits(31, 32) and genomic features, using area under the receiver operating characteristic (AUROC) to evaluate performance. A model with AUROC of 0.5 has no predictive ability, whereas a model with AUROC of 1.0 has perfect predictive performance. We selected predictive variables from among 13 genome-wide summary statistics including demographic history, genetic diversity, and genetic load variables, ~57,000 window-based metrics per genome, and 39 ecological variables from PanTHERIA(15) including physiological, life-history, and behavioral variables (table S4). Models including only genomic features and no ecological variables (17 models; AUROC ranged from 0.69–0.82) performed worse than models including only ecological variables (1 model; AUROC 0.88) and similarly to models including both genomic and ecological variables (17 models; AUROC range 0.68–0.83; table S5). Models with only genomic features were, however, consistently better able to distinguish threatened from non-threatened species (tables S5–S6; fig. S11–13) compared to random chance (i.e. AUROC of 0.5). Models including only genomic variables performed similarly to other studies that predicted IUCN status from ecological or morphological data with comparable sample sizes (e.g. AUC ranging from 0.67–0.90 for n=171–430 species) (33–35).

The number of species with values for ecological, genome-wide summary statistics, and window-based metrics differed, which may affect model performance. To compare the predictive value of genomic and ecological features directly, we next tested models in a set of 210 species for which both data types were available (tables S4 and S6). Again, the model with genome-wide summary statistics alone was predictive of threatened status (AUROC 0.71), but performed more poorly than the model with ecological variables (AUROC 0.83). Combining genomic summary statistics with ecological variables led to a modest improvement in distinguishing threatened from non-threatened species (AUROC=0.85) compared to genomic variables alone, with *N*_*e*_ as the fourth most important predictor in the model after weaning age, age at first birth, and age of sexual maturity (fig. S14). Models including genomic window-based features never outperformed models with ecological variables alone (table S6), suggesting that complementary information provided by genomic versus ecological data may be better captured by summary or transformed variables (e.g. principal components) than by numerous weakly informative window features that may overwhelm the predictive models. Overall, our evaluation suggests that while genomic information from a single individual is not better than ecological data for predicting threatened status, these data do have predictive value, especially when ecological variables are unavailable.

As a demonstration of their utility, we applied our regression and random forest models to predict the status of three species considered “Data Deficient” by the IUCN (Fig. 3D). The models suggest the Upper Galilee Mountains blind mole rat (*Nannospalax galili*), which lacks ecological data, is least likely to be threatened (11–44% probability), whereas the killer whale (*Orcinus orca*), for which both ecological and genomic data are available, is more likely to be threatened (35–68% probability), consistent with the identification of some at-risk populations(36). Predictions for the Java lesser chevrotain (*Tragulus javanicus*) depend on model specifications, with the highest threat prediction from the within-order regression model (67% probability), and other models suggesting it is less likely to be threatened (24–49% probability). The results indicate that, among the three species, the killer whale should be prioritized for further study, and demonstrate how genomic data can provide a rapid and inexpensive initial conservation assessment.

## Discussion

Our results provide empirical support for theoretical predictions that small populations accumulate and fix weakly and moderately deleterious alleles, and demonstrate a correlation between historical effective population size and contemporary extinction risk. We found little evidence, however, that species with historically small effective population sizes have higher risks of extinction because of elevated drift load. Alternatively, historically small populations may have elevated extinction risk simply because these populations are small and thus more vulnerable to other threats such as habitat loss or change, the introduction of infectious disease, competition with invasive species, and new hunting or predation pressures.

Despite the limitations of assuming that a single genome is representative of the diversity within a species, our comparative genomics approach allowed us to maximize the number of species analyzed to explore the power to detect genomic correlates of endangerment. Empirical studies suggest a single individual can represent a species for characteristics shaped by long-term evolutionary history; variation in the proportion of deleterious mutations is typically smaller within species than between(37, 29), and historical *N*_*e*_ estimates are consistent across conspecifics(38, 39). The analysis of multiple resequenced individuals per species, however, will increase accuracy and resolution by capturing intraspecific variation in genetic diversity, heterozygosity, and inbreeding (especially for species with strong population structure), enabling estimation of allele frequencies, improving inference of more recent demographic history, and allowing better detection of rare and segregating variants(e.g. inbreeding load; *22*). The latter may be particularly important for estimating extinction risk, as segregating variants tend to be enriched for deleterious alleles(40, 41) and may disproportionately impact extinction risk from population bottlenecks(12). In the future, larger data sets comprising multiple individuals per species may shed light on long-standing questions about the relative impact on fitness of many weakly deleterious alleles versus a few strongly deleterious alleles(22, 25, 37, 42, 43).

Inferring real-world fitness from genomic data includes caveats. Evolutionary constraint may, for example, reflect past selection on loci that no longer impact fitness(44). Loci that seem functionally important in model species may be irrelevant to the species of interest, compensatory mutations may ameliorate the impact of deleterious mutations, and factors such as dominance, epistasis, pleiotropy, and purging may also complicate the relationship between genetic load and fitness. Finally, local differences in habitat may mean that the impact of deleterious mutations differs among individuals or populations(25, 45, 46). For these reasons, the impact of the observed proportionally higher load in smaller populations will be challenging to know in the absence of direct fitness data, such as reproductive success and the frequencies of genetic diseases and congenital abnormalities(26, 43, 47).

As additional genomes and population resequencing data become available(48), the power and accuracy of predictions of extinction risk from genomes will improve(8). Our analyses of the genomes of single individuals, which can be generated rapidly and inexpensively(49), demonstrate the potential for using genomic estimates of demography, diversity, and genetic load to triage species in need of immediate management intervention, and we join in the calls for including genomics into conservation status assessments(50–53).

## Materials and Methods

We provide a summary of our materials and methods below; refer to the Supplemental Materials and Methods for further detail.

### Mammal genomes and metadata

We examined genomic variation in 240 species represented by 241 reference genomes in the Zoonomia multispecies alignment. The genome assemblies varied in quality, with contig N50 values ranging from 1 KB to 56 MB (table S1). Short-read sequence data, usually from the reference individual, were used to estimate metrics related to historical demography, heterozygosity, and heterozygous deleterious variants from single genomes. Homozygous deleterious genetic load was estimated relative to reconstructed ancestral sequences from the multispecies alignment (fig. S1). We tested correlations between all genomic metrics, and between genomic metrics and extinction risk, using a statistical framework that accounts for phylogenetic relationships across species. Using regression and machine learning models, we tested the potential for genomic data to predict the conservation status of species.

For all species, we compiled metadata on conservation status, diet, and generation time (table S1). We assigned a conservation status (Least Concern (LC), Near Threatened (NT), Vulnerable (VU), Endangered (EN) or Critically Endangered (CR)) to the lowest known taxonomic level of the sequenced sample, using the IUCN Red List of Threatened Species (IUCN Red List API v. 3) as a proxy for extinction risk. We classified each species as carnivore, herbivore, or omnivore based on(54), using information for the genus when species-specific information was unavailable. From available metadata, we categorized the sample used for both the reference genome and short-read data as a wild, captive, or domesticated individual.

Tests for correlations between variables were conducted with phylogenetic linear regression or phylogenetic logistic regression in the R package *phylolm(55)*, incorporating the phylogenetic tree with branch lengths(56) to account for non-independence.

### Estimating historical effective population sizes and genome-wide heterozygosity

We called heterozygous positions in all genomes with short-read data using the GATK best practices pipeline as described previously(7). Briefly, we mapped paired-end sequencing data to the respective genome assemblies using BWA mem (version 0.7.15)(57), marked and removed optical duplicates, and called heterozygous variants using the HaplotypeCaller module of the GATK software suite (version 3.6)(58).

We inferred the history of effective population sizes (*N*_*e*_) for each species using PSMC (version 0.6.5-r67)(59). We called variants in each genome from scaffolds >50KB in length, filtered for sequence read coverage and base quality score, and used these as input for PSMC. We rescaled the PSMC output using species-specific generation times(60) and a mammalian mutation rate(21) and calculated the harmonic mean across temporal estimates from periods >10 kya. To compare contemporary population sizes to historical N_e_, we obtained census population estimates (*N*_*c*_) for 89 species from the PanTHERIA database(15), estimating *N*_*c*_ as the product of population density and geographic area from census data(15, 61).

To identify runs of homozygosity (RoH), we used our previously described method(7). For every assembly, we calculated the ratio of heterozygous to callable positions in non-overlapping, 50-kb windows, and fit a 2-component Gaussian Mixture Model to the joint distribution, which is expected to be bimodal with a peak at the lower tail of the distribution corresponding to runs of homozygosity (fig. S1B). Windows were then assigned as RoH or non-RoH and used to calculate the proportion of the genome in RoH (fRoH), genome-wide heterozygosity, and outbred heterozygosity (i.e. heterozygosity in non-RoH regions; figs. S2 and S15).

### Deleterious genetic load

We called heterozygous variants from single sample, short-read data mapped to the reference genome of each species. Homozygous substitutions were estimated from each reference genome relative to the closest reconstructed ancestral sequence in the phylogeny using the *halBranchMutations* tool in the Comparative Genomics Toolkit(62). Because new alleles become fixed or lost on the order of <4*N*_*e*_ generations(63), most homozygous substitutions between species are likely fixed. We assessed the potential functional impact of mutations by 1) evolutionary conservation of the site (phyloP), and 2) the estimated impact of the mutation on protein-coding genes. Mutations at evolutionarily conserved sites (phyloP>2.27;(9)), and those that cause nonsynonymous changes in protein-coding genes, were assumed to be predominantly harmful(19). Variant sites in each genome were assigned human-based phyloP scores estimated from the multispecies alignment(9). To infer functional impacts on protein-coding genes, each genome was annotated with human orthologs by lifting over human exon intervals to the target species. Synonymous, missense and loss-of-function variants were then estimated in the program *SnpEff* v.5.0e(64). We also examined mutations in single-copy genes with associated viability phenotypic data in knockout mice as classified by the International Mouse Phenotyping Consortium (IMPC)(23), using IMPC categories (e.g. lethal or viable) as a proxies for gene essentiality and the potential fitness impacts of mutations in these genes(23).

### Predicting threat from genomic variables

To predict whether a species is threatened (NT, VU, EN, and CR categories) or non-threatened (LC category), we modeled conservation status across species from genomic variables using both regression and machine learning models.

We took two main approaches in our regression models of conservation status across species, using 1) phylogenetic logistic regression to model threatened versus non-threatened status, which allowed us to test the significance of predictor variables, but not make predictions for species with unknown threat status, and 2) ordinal regression models of specific IUCN categories, which allowed us to test significance and make predictions for species with unknown threat status. Unlike logistic regression, ordinal regression did not inherently incorporate the phylogeny, so we included taxonomic order as a factor in the models. We tested 13 genomic variables (table S2), modeled individually and as principal components, and included taxonomic order and dietary trophic level, a previously described correlate of extinction risk(65). We estimated model error by fitting parameters on 80% of the data and testing the remaining 20% of the data across 100 runs with different data subsets.

We used random-forest based classification to estimate the likelihood that a species is threatened from 13 genome-wide summary statistics of heterozygosity, demographic history, and genetic load, and from 5 genomic metrics within homologous 50KB windows (table S4). We trained models using the two genomic data types (windows-based and genome-wide) separately and combined, and incorporated 39 ecological variables from the PanTHERIA database (table S4). We used the scikit-learn 1.0.2 package for fitting all the models(66).

We first split our dataset into a 75% training set and a 25% test set. For each model, we performed preprocessing and imputation steps using only the training data, then trained the model on the training set and evaluated it on the test set. We ran 5-fold cross validation on the training set to determine the optimal set of hyperparameters, tuning the number of decision trees, the maximum depth of the trees, and the number of features used at each decision to optimize a performance metric. We used AUROC to estimate how well a model predicts the correct output class. AUROC is designed to be more robust to class imbalance in comparison to a metric such as accuracy.

To leverage all available data, we first ran models using all species with data for a given data type (table S5). The number of species with values for ecological, genome-wide summary statistics, and window-based metrics differed however, which may impact the results. To compare the performance of ecological and genomic variables and their combination across the same set of species, we also trained and tested models in the set of species for which both data types were available (table S6).

The Zoonomia alignment included three species classified as “Data Deficient” by the IUCN, the Upper Galilee Mountains blind mole rat (*Nannospalax galili*), the Java lesser chevrotain (*Tragulus javanicus*), and the killer whale (*Orcinus orca*). The blind mole rat lacked ecological data on PanTHERIA. We used the within-order and across-order ordinal regression models and all random forest models to predict the probability that these species are threatened.

## Supplementary Material

supplement Figs and Tables S1-4

tables S5 and S6

Table S1

## Data Availability

The data presented in this paper are detailed in supplementary materials. Summary data and analysis scripts are available at https://github.com/LaMariposa/zoonomia_biodiversity. NCBI accession numbers for sequence data used in analyses are given in table S1.
