# Supplementary material for: The contribution of historical processes to contemporary extinction risk in placental mammals
Source: Science. Author manuscript; Available in PMC 2023 May 15. (PMC10184782; doi:10.1126/science.abn5856)
Supplement: supplement Figs and Tables S1-4 [file NIHMS1893031-supplement-supplement_Figs_and_Tables_S1-4.pdf]

Materials and Methods

We examined variation in 241 reference genomes from 240 species. Each species was represented by a single genome, with the exception of *Canis lupus*, which was represented by two genomes—one domestic breed and one village dog. The reference genomes varied in quality, with contig N50 values ranging from 1,039 to 56,413,054, with a median of 45,189 (table S1). For some of these species, no short-read sequencing data were available from NCBI to map to the reference genome (n=8), variant calling failed (n=11), or downstream pipelines failed (heterozygosity-related metrics, n=13, PSMC, n=12). The reference genomes were used to estimate homozygous deleterious genetic load; while the short-read sequence data (usually from the reference individual) were used to estimate metrics related to historical demography, heterozygosity, and heterozygous deleterious variants (fig. S1). We examined correlations between these metrics using statistical methods that account for relationships across the phylogeny, and examined genomic features of extinction risk to predict the conservation status of species.

Metadata

We compiled metadata on conservation status, diet, and generation time for the 240 placental mammal species in the Zoonomia alignment (table S1). We determined the conservation status (Least Concern (LC), Near Threatened (NT), Vulnerable (VU), Endangered (EN) or Critically Endangered (CR)) and population trends (declining, stable or increasing) using the IUCN Red List category (IUCN Red List API v. 3) based on the scientific name of the species. We use IUCN category as a proxy for extinction risk, however we recognize that because the assessments are often done at the species level, the categorization of a species may miss important variation between populations. Where we were able to determine a specific subspecies or population for the sequenced sample, we used the IUCN category for the lower taxonomic level. For the diet category, we classified each species as either carnivore, herbivore, or omnivore based on (*54*). In cases where species-specific diet information was unavailable, we used data reported at the genus level. We categorized as carnivores all species for which other animals made up a majority of their diets, including terrestrial vertebrate-eaters, insectivores, piscivores, and planktivores; we also considered the vampire bat, a hematophage, to be a carnivore. Any animal with a diet composed of both plant products and animal products was considered an omnivore. Species for which the diet was all or nearly all plant products were considered herbivores; some of these species consumed insects occasionally or as a minor part of their diets. For generation time, we used a published database of mammalian generation lengths (*60*). If a species was not in the database, we used the value from the next closest species. We determined species specific mutation rates per generation by multiplying an average mammal mutation rate of 2.2e-9 basepairs per year (*21*) by the species-specific generation time in years.

We compiled additional metadata associated with provenance of the specific sample that was sequenced for the genome of each species. For 39 samples used for reference genome assembly, there were no publicly available short-read Illumina sequencing data, which were necessary for analyses based on heterozygous sites (i.e. heterozygosity, segments of homozygosity, heterozygous deleterious variants, and PSMC). For 31 of these we identified an alternate sample with resequencing data, choosing a sample as similar to the reference individual as possible (e.g. from the same population). For each sample (including both reference genome samples and, if different, short-read data samples), we determined subspecies or population

information and whether the sample was a wild (including captive offspring of wild-born parents), captive, or domesticated individual. We obtained sample information from the NCBI records and published papers that used the sample, such as the genome announcement papers. In some instances, insufficient metadata were available, but informal project summaries provided details. For 16 samples, no additional data were available and the sample metadata were marked as unknown.

Alignment and variant calling of short-read sequencing data

We interrogated the assemblies included in the Zoonomia alignment for heterozygous positions using the GATK best practices pipeline as described previously (*7*). We removed adapters with Cutadapt (version 1.10)(*67*). This step was not done for the alignments used for PSMC analysis for the original Zoonomia genomes (*7*), but this difference should not affect our results since the alignment algorithm soft clips reads (*57*). We mapped the paired-end sequencing data corresponding to each assembly against their respective assemblies using BWA mem (version 0.7.15)(*57*). We marked and removed optical duplicates using the PICARD MarkDuplicates tool (version 2.5.0)(*68*). Finally, we called heterozygous variants using the Haplotypecaller module of the GATK software suite (version 3.6)(*58*).

Phylogenetic regression

All regressions of variables across species were conducted with phylogenetic linear regression or phylogenetic logistic regression in the R package *phylolm* (*55*), incorporating the phylogenetic tree with branch lengths (*56*) to account for non-independence. Where we report means for groups compared in phylogenetic regressions, we report the phylogenetically-adjusted means.

Dynamics of historical effective population sizes

We inferred the history of effective population sizes (N_e) for each species using PSMC (version 0.6.5-r67)(*59*). We used the short-read alignments generated for variant calling of scaffolds greater than 50 kb (*69*). For each alignment, we used samtools depth (version 1.11-3-g7028dd4)(*70*) to determine the average depth of coverage. To prepare for PSMC, we generated a pileup file with samtools mpileup (version 1.7)(*70*) from the 50-kb alignment files, retaining anomalous read pairs (-A) and downgrading mapping quality for reads with excessive mismatches (-C50). We then called variants on the pileup file using bcftools call (version 1.8)(*71*), using the consensus caller (-c). From the variant file we generated a consensus fastq file using vcfutils vcf2fq (version 2014)(*70*), with a minimum coverage of one-third the sample's average coverage and a maximum coverage of two times the sample's average coverage. We then generated a PSMC input fasta file using the PSMC's fq2psmcfa with a minimum base quality score of 20 (-q20). Finally, we ran PSMC with default parameters, except we altered the parameter intervals to -p "4+25*2+4+6", as suggested for humans (*72*). We rescaled the output of PSMC using the species-specific generation times and mutation rates (see *Metadata* section).

To estimate historical N_e, we calculated the harmonic mean from the PSMC estimates of effective population size through time, excluding time intervals less than 10 kya. While our samples varied in level of inbreeding, we do not expect this to have a substantial impact on our estimates of historical N_e. Previous work examining inbred samples showed similar PSMC curves regardless of whether runs of homozygosity were included or excluded in the analysis (*38*, *73*). Additionally, our genomic data varied in other important aspects, including genome

quality and coverage, and PSMC has been shown to be robust to variation in these characteristics (*39*, *74*, *75*).

Inferring recent population declines from N_e/N_c ratios

To compare contemporary population sizes to historical N_e, we obtained census population estimates (N_c) for 89 species from the PanTHERIA database (*15*), estimating N_c as the product of population density and geographic area from census data (*15*, *61*). Although not a true population census, it provides an overall gauge of the potential number of individuals within a species' current distribution. N_c estimates ranged widely across species, from 2,909 (*Bison bison*) to 65,971,017,419 (*Procavia capensis*). Although the values are not meant to be interpreted as real census population sizes, they provide a gauge of relative census population sizes across species. As expected, N_c was strongly correlated with IUCN status (phylolm, $mean_{threatened}$=16,619,347; $mean_{non-threatened}$=90,341,802; p=6.1e-7), as it is a criterion for IUCN status assessments, but examining N_e/N_c ratios can nonetheless provide additional information on recent declines not reflected in the genome. Species with larger N_e/N_c ratios were slightly more likely to have "declining" population trends classified by the IUCN Redlist than "stable" or "increasing" (phyloglm, β= 0.59, p=0.026, where each 10-fold increase in N_e/N_c increases odds of being categorized as declining by 59%), suggesting that N_e/N_c may be useful for identifying recent declines. N_e/N_c ratios are influenced by life-history traits, including mating strategy, range size, trophic level, generation time, population structure and population fluctuations (*14*, *76*), but we nonetheless found a comparable relationship between N_e/N_c and conservation status within Primates (phylolm, $mean_{threatened}$=3.46e-3; $mean_{non-threatened}$=1.11e-3; p=0.029), the only group with enough N_e/N_c estimates in both threat categories, suggesting that it is not driven by life-history traits alone. Because N_e/N_c ratios require population census information, and thus it is not useful for informing conservation status of species that lack this information, N_e/N_c may nonetheless be valuable for identifying species with historically large populations that have recently declined.

Estimating runs of homozygosity (RoH) and heterozygosity

We used an identical strategy to the Zoonomia data release paper to identify runs of homozygosity (RoH) (*7*). Briefly, for every assembly, we calculated the ratio of heterozygous positions per callable base pair in non-overlapping, 50-kb windows. Then, we used the pomegranate python environment (*77*) to fit a 2-component Gaussian Mixture Model (with a third component to capture outliers and low confidence windows, such as windows with large amounts of missing data) to the joint distribution of all heterozygosity windows in the assembly. These joint distributions are expected to be bimodal, with a sharp peak at the lower tail of the distribution corresponding to low heterozygosity regions, such as runs of homozygosity. Finally, each window was assigned to its most likely component (RoH or non-RoH) based on the model's posterior probabilities. We note that this method is unable to distinguish between true segments of homozygosity and other genomic regions with very low heterozygosity. However, given the large window size, we expect that only a small proportion of the genome would be miscalled due to low heterozygosity. Additionally, all species are likely to be impacted by this minor bias, so we do not expect it to substantially affect relative estimates of metrics using RoH.

For each species, from the windows assigned as either heterozygous or homozygous we calculated the proportion of the genome in RoH (fRoH) (fig. S15), genome-wide heterozygosity, and outbred heterozygosity. To estimate fRoH we calculated the length of the genome in RoH

and divided it by the total length of the genome assigned as either RoH or non-RoH. We next calculated genome-wide heterozygosity as the mean of the heterozygosity estimates from the 50-kb windows, weighted by the length of the segment to account for the shorter segments at the ends of scaffolds. Lastly, we estimated heterozygosity in non-RoH regions (i.e. outbred heterozygosity) using the mode of the distribution of 50-kb window heterozygosity estimates with regions identified as RoH excluded. To ensure the accuracy of the estimation of the mode of the non-RoH distributions, we manually inspected both the full distribution and the non-RoH distribution. In 25 instances, we needed to correct the automated call. One scenario where this occurred was when the full distribution was not bimodal due to low overall heterozygosity and the mode of the distribution was zero. For these, we examined the full distribution and set heterozygosity to the peak when most windows were non-RoH. The second scenario where automated calls needed to be corrected was when the distribution of the non-RoH segments was bimodal due to miscalled RoH. In these instances, we were able to visually identify a clear non-RoH peak in the distribution.

Adding neutral diversity statistics for 79 additional species relative to our previous analysis (*7*), we substantiate the result that species with threatened IUCN status had, on average, significantly lower genome-wide heterozygosity (phylolm, $mean_{threatened}$=0.0024, $mean_{non-threatened}$=0.0029, p=0.017; fig. S2). However, unlike the previous results, we found that the proportion of the genome in RoH (fRoH) was highly variable across the expanded dataset (fig. S15), and the mean was actually lower for threatened species compared to non-threatened species (phylolm, $mean_{threatened}$=0.21, $mean_{non-threatened}$=0.27, p=0.015). This contrasting result is likely due to the skewed distribution of RoH (fig. S15). Furthermore, intraspecific variation in heterozygosity and fRoH, which is not captured in our data because we used a single individual from each species, may add variability that makes any correlation with endangerment status more difficult to detect.

Estimating deleterious genetic load

We estimated homozygous substitutions from the reference genome sequences, calling derived substitutions relative to the most recent ancestral sequence in the multispecies alignment. Reconstructed ancestral sequences are included in the multispecies alignment HAL file (https://cglgenomics.ucsc.edu/data/cactus/) that was previously generated using the program Progressive Cactus (*7*), which implements ancestral reconstruction for all nodes in the multiple alignment procedure by incorporating multiple ingroup and outgroup sequences (*78*). We used the *halBranchMutations* tool in the Comparative Genomics Toolkit (*62*), which annotates the locations of single nucleotide substitutions on a branch-by-branch basis relative to the closest ancestral node, thus calling derived substitutions arising along the branch for each species in the alignment. We assume that most of these substitutions are likely to be fixed because typically enough time has elapsed since the ancestral node for the derived alleles to have become fixed or lost (on the order of $<4N_e$ generations)(*63*). We found that genomic windows that aligned to multiple regions of the genome tended to have many substitutions. Because querying the multispecies alignment HAL file to directly identify regions with duplicate alignments is very computationally expensive, we filtered regions with more substitutions than expected from a poisson distribution, though this step likely excludes true hypervariable regions from our analysis. Therefore, we filtered potentially spurious calls by fitting the number of substitutions in all 1KB windows across the genome to a poisson distribution, and removing windows identified as outliers at alpha=0.1 using the function aout.pois in the R package *alphaOutlier*

(https://CRAN.R-project.org/package=alphaOutlier). Heterozygous variants (which have, by definition, one derived and one ancestral allele) were identified from single sample, short-read data mapped to the reference genome of each species as described above. We included in our analysis only single nucleotide polymorphisms, heterozygous sites with genotype quality (GQ; the Phred-scaled confidence that the genotype assignment is correct)>80, and read depth (DP) < three standard deviations from the mean DP across variant sites for a given sample.

To assess the functional impact of each derived mutation, we used 1) evolutionary conservation at the site, and 2) the estimated impact of the mutation on protein-coding genes. First, for evolutionary conservation we assigned human-based conservation $-\log_{10}$ p-values (phyloP scores) estimated by the Zoonomia consortium (*9*). Briefly, the PHAST v1.5 package: https://github.com/CshlSiepelLab/phast (*79*) was used to estimate phyloP scores under a null hypothesis of neutral evolution, performing a likelihood ratio test at each alignment column (--method LRT) of the human-referenced, 241-way, duplicate-filtered alignment. To assign these phyloP scores to derived mutations identified in each genome, we lifted over all derived mutations to the human genome using *halLiftover* and the 241-way mammalian alignment, ignoring paralogous alignments using the --noDupes option (*62*). We were specifically interested in evolutionarily conserved sites which have a positive phyloP score, and thus to minimize the influence of negative phyloP scores that reflect accelerated evolution (*80*), we set all negative phyloP values to 0. We noted differences in genome-wide phyloP scores across taxonomic orders. To determine whether the differences could stem from using human-based phyloP scores, we also assigned phyloP scores derived from mouse and dog genomes to heterozygous sites for a subset of 115 genomes from across the phylogeny. Mean phyloP scores from human, mouse and dog were highly correlated (r^2>0.99), indicating no substantial bias stemming from the genome used as the basis for phyloP scores. Furthermore, in tests that account for phylogenetic relationships (phylolm), mean phyloP scores did not significantly differ across taxonomic orders, suggesting that phylogenetic regressions adequately account for variation across orders. Specifically, taxonomic order did not explain mean phylop across substitutions better than intercept-only phylogenetic regression models (run with the phylostep function of phylolm), suggesting that the significant relationships between phyloP and other variables identified using these methods were not driven by the phylogeny.

Second, we inferred functional impacts from genome-specific gene annotations. Genes were estimated by lifting over human annotated transcripts through genomes in the alignment via *halLiftover* (*81*). Briefly, the human exon intervals were lifted over to the target species, and for each exon the resulting range was consolidated into a single range per contig with 500 bp added to both ends. The target sequence within the resulting interval was then aligned to the human protein sequence using *exonerate* (*82*), keeping only the best alignment. The alignment was checked to make sure that it resulted in a contiguous reading frame, that the predicted protein started with methionine, and that the predicted protein was within 90-110% of the length of the human reference protein. Using these gene annotations for each genome, we estimated synonymous, missense and loss-of-function (LoF) variants using the program *SnpEff* v.5.0e with default settings (*64*). *SnpEff* defines LoF variants as those causing complete loss of function of the affected transcripts: stop codon-introducing (nonsense) or splice site-disrupting single nucleotide variants predicted to disrupt a transcript's reading frame, affecting more than 50% of the protein-coding sequence. For homozygous sites, the effect of the ancestral allele was predicted relative to the focal genome, and thus homozygous LoF substitutions could not be reliably called, and we instead focused only on missense substitutions for homozygous sites. We

assumed that mutations at sites that are more conserved, that cause missense and LoF changes in protein-coding genes, especially those that show lethality as a result of LoF, are more likely to be harmful and to contribute to genome-wide deleterious genetic load (*19*).

Given these assumptions, we measured homozygous genetic load as the distribution of phyloP scores across all homozygous substitutions (mean and Pearson's kurtosis estimated from the *moments* package in R), the proportion of homozygous substitutions in protein-coding genes that are missense, and the proportion of homozygous substitutions that are at an evolutionarily conserved site (phyloP>2.27; (*81*)). Because homozygous substitutions were estimated for each species relative to the closest ancestral node in the phylogeny, the number of substitutions depended on the distance to the nearest species in the dataset, and ranged from 145,602 for *Cavia tschudii* (montane guinea pig) to 53,919,964 substitutions for *Hystrix cristata* (crested porcupine). There was a negative correlation between the proportion of putatively deleterious substitutions and the distance between a species and its closest ancestor (e.g. log-log linear regression r^2=0.415 for missense substitutions at conserved sites). Comparisons between closely related species are typically enriched for nonsynonymous substitutions relative to more distant species (*41*). To adjust for this bias, we performed log-log linear regressions of homozygous genetic load variables against the total number of substitutions, and for variables that were significantly correlated (proportion of missense substitutions, proportion of conserved substitutions, proportion of missense substitutions at conserved sites, and kurtosis of phyloP), we used the residuals for subsequent statistical tests of the relationship between genetic load and demographic variables. We reported p-values for the branch-length adjusted variables, and presented the non-adjusted values and their coefficients in figures for readability and interpretability. For phylogenetically corrected logistic regression tests (phyloglm), we present coefficients (β) converted to the change in odds of being threatened using e^{β}-1.

To further parse the potential fitness consequences of mutations, we estimated the proportion of homozygous missense, heterozygous missense and heterozygous LoF single nucleotide mutations in genes that differ in their essentiality (i.e. the requirement of a gene for an organism's survival). We limited the analysis to single-copy genes with associated viability phenotypic data in knockout mice as classified by the International Mouse Phenotyping Consortium (IMPC) (*23*). From all genes (annotated with human orthologs as described above), we selected single-copy genes using the BUSCO mammalia_odb10 dataset, searching pub_og_id names against the OrthoDB v.10 database, and retaining hits identified as single-copy in >90% of the mammalian species set. The IMPC set of genes with a viability phenotype (Data Release 15.0) is provided for one-to-one mouse-human orthologs, which achieve an agreement support score of at least 5 out of 12 of the inference methods implemented by HGNC Comparison of Orthology Predictions (HCOP; i.e., support>=5, one-to-one in both directions, human to mouse and mouse to human). Single-gene knockout mouse lines are assigned a lethal, subviable or viable phenotype category based on the observed number of viable homozygote pups at pre-weaning stage. These categories can be used as a proxy for gene essentiality and, consequently, for the potential fitness impacts of mutations in these genes (*23*). The number of genes in each category varied across species depending on the completeness of the annotation for that genome. The IMPC lethal category had on average 263 genes annotated in each genome (range 19-782), and the IMPC viable category had on average 530 genes (range 40-1564). Because there were relatively few genes in the IMPC subviable category, and the results from the subviable category were qualitatively similar to the lethal category, we presented results from only the viable and lethal gene categories in the main text. To minimize noise associated with estimation of

heterozygous variants from low sequencing depths, we restricted the analysis to 131 genomes with mean read depth >= 20x and mean genotype quality (GQ; the Phred-scaled confidence that the genotype assignment (GT) is correct) >= 80 across heterozygous sites. For homozygous substitutions, we restricted the analysis to 220 genomes with >=10,000 substitutions in coding regions. We evaluated both missense and LoF variants at heterozygous sites, and missense substitutions for homozygous sites. From the genome of a single individual, we are likely to capture many thousands of mildly and moderately deleterious alleles that are at high frequency or fixed (drift load), but only a few highly deleterious/lethal alleles (which are typically rare in the population and across the genome, and comprise mainly inbreeding load); thus we likely do not have high power to detect differences in highly deleterious alleles between species.

Correlations between demography, genetic diversity, genetic load and conservation status

Species with smaller historical effective population sizes tend to have higher proportions of mildly to moderately deleterious mutations in their genomes. The proportion of homozygous substitutions at conserved sites was negatively correlated with species N_e (phylolm, p=9.65e-3, β= -1.14e-3, where each 10-fold increase in N_e corresponds to a 1.14e-3 decrease in the proportion, fig. S4A), and the proportion of homozygous missense substitutions was negatively correlated with species N_e (phylolm, β= -0.020, p=7.76e-5; fig. S4B). Phylop kurtosis (which describes the extremity of phyloP outliers in the tail of the distribution across substitutions) was positively correlated with N_e (phylolm, β=0.851, p=0.014), i.e. species with smaller N_e had smaller right tails, suggesting fewer extreme conservation scores. In contrast to historical N_e, neither genome-wide heterozygosity nor the proportion of the genome in RoH (metrics that are influenced by more recent population history) were significantly correlated with the proportion of deleterious variation in the genome (phylolm, all p>0.098).

We then parsed the potential fitness impacts of mutations by examining genes classified as having lethal, subviable and viable phenotypes in knockout mice (figs. S5-S6). As expected for genes under strong purifying selection, there were proportionally fewer missense variants in subviable and lethal gene categories compared to genes in the viable category across species (ANOVA, all p<2e-16, fig. S7), validating the relative impacts of mutations in genes inferred from IMPC categories. The historical N_e of species was negatively correlated with the proportion of heterozygous missense variants for all IMPC categories (phylolm, all p< 2.53e-3; fig. S5), and with homozygous missense substitutions in the viable and lethal categories and in all gene categories combined (phylolm, all p< 1.72e-5; fig. S5). By contrast, heterozygous LoF variants were not significantly associated with N_e, except for a negative correlation for LoF alleles in IMPC lethal genes (phylolm, p=0.019; fig. S5), and the proportion of LoF alleles did not significantly differ between threatened and non-threatened species (fig. S6). Because of the rarity of LoF alleles in the genome, we had little power to test for differences in LoF alleles across species. To assess whether differences in annotation impacted our LoF results, we reran the regression with N_e using LoF estimates only for species with at least 200 genes in the IMPC lethal and viable categories and found that the results did not qualitatively change. Populations with smaller N_e had larger variability in the proportion of LoF alleles, especially in genes in the IMPC lethal category (fig. S5); however, the overall number of heterozygous sites is lower in these species, which adds additional stochasticity to the estimates.

While we do find that species with small N_e have proportionally higher genetic load, species with large N_e are expected to have more deleterious alleles segregating at low frequency by count

(*12*, *22*). We examined heterozygous deleterious variants, normalized by the number of genes annotated in each genome, and found that that species with larger N_e have more heterozygous missense variants in IMPC viable genes (log-log phylolm, β=0.222, p=0.002) and IMPC lethal genes (log-log phylolm, β=0.167, p=0.03), as expected from theory.

Deleterious genetic load in threatened compared to non-threatened species was often, but not always, consistent with expectations for small compared to large populations, respectively. Phylop kurtosis was lower on average in threatened than non-threatened species (phylolm, $mean_{threatened}$=22.03, $mean_{non\text{-}threatened}$=22.75, p=0.001), a trend largely driven by Carnivora (phylolm, $mean_{threatened}$=24.39, $mean_{non\text{-}threatened}$=25.95, p=0.047) and Primates (phylolm, $mean_{threatened}$=23.96, $mean_{non\text{-}threatened}$=25.32, p=7.9e-4)(fig. S8). There was no significant difference in phylogenetically corrected means of proportional genetic load between threatened and non-threatened species, including the proportion of missense substitutions (phylolm, p=0.31), the proportion of substitutions at conserved sites (phylolm, p=0.46), and the proportion of missense substitutions at conserved sites (phylolm, p=0.53)).

There were significant relationships between fixed genetic load and the odds of being threatened, however, the relationship was different for protein-coding genes compared to evolutionarily conserved sites genome-wide. Species that had proportionally fewer homozygous substitutions at evolutionarily conserved sites across the genome were more likely to be threatened in logistic regression tests (phyloglm, β= -0.52, where each 1% increase in these substitutions is associated with a 52% decrease in odds of being threatened; p=1.38e-05; fig. S4C), even though species with smaller N_e tended to have proportionally more homozygous substitutions at conserved sites (phylolm, p=9.6e-3; fig. S4A). Species with lower kurtosis of the phyloP distribution across substitutions (i.e. fewer extremely conserved outliers) were also more likely to be threatened (phyloglm, β= -0.17, p=0.018, fig. S8). In protein coding regions, by contrast, species with proportionally more missense substitutions were more likely to be threatened (phyloglm, β=0.23, where each 1% increase in these substitutions is associated with a 23% increase in odds of being threatened; p=0.002; fig. S4D). Genomes with proportionally more missense substitutions in IMPC categorized genes were also more likely to be those of threatened species for nearly all gene categories (phyloglm, all p<0.053; fig. S6).

Impact of variation in annotation performance across species

The number of genes annotated in each genome across species varied widely (range=1760-8465, mean=5992, std. dev.=1311), with Primate genomes having the most genes annotated. However, because we estimated genetic load as the proportion of deleterious mutations relative to total coding mutations (and not by counts of deleterious mutations), there was not a strong effect of different numbers of annotated genes used in the analysis. To determine whether differences in annotation performance may have impacted our results, we estimated the proportion of missense substitutions using only the subset of genes that were annotated in at least 200 species. The results were very similar and qualitatively identical. The estimated proportion of missense substitutions for species using the restricted and full sets of single-copy BUSCO genes were highly correlated (r^2=0.94). The significance of the relationships between the proportion of homozygous missense substitutions and threatened status (phyloglm, β= 0.20, p=0.013), and between the proportion of homozygous missense substitutions and N_e (phylolm, β= -0.019, p=1.72e-5) were also qualitatively identical with the more restricted set of genes relative to the full set.

Additional lines of evidence suggest that overall our estimates of genetic load are robust. 1) The observation of proportionally fewer deleterious mutations with increasing N_e fits theoretical expectations that purifying selection is more effective at removing/reducing deleterious alleles in large populations, and confirms that our classification of deleterious mutations is correlated with the true deleterious fitness impacts across mutations. 2) Mutations and derived alleles were estimated using distinct methods for homozygous versus heterozygous sites. (Homozygous derived substitutions were called relative to ancestral reconstructions from the multispecies alignment, and their impact inferred from evolutionary conservation and/or changes to protein coding sequences, whereas heterozygous variants were called from short-read data mapped to reference genomes, and a single derived allele was assumed.) Yet there are negative correlations between N_e and proportional genetic load for both mutation types (Fig. 2), which further supports our classification of deleterious alleles.

Using single genomes to represent genetic load of a species

While a single genome can never encompass intraspecific variation, by using a single genome per species we were able to include species that had minimal genomic resources and increase the number of species analyzed. The proportions of deleterious mutations are driven by the effects of purifying selection to remove these variants and the effects of genetic drift over time, and thus we expect that individuals within a species should have similar proportions of genetic load, and these proportions would not rapidly change with, for example, recent changes in demography. For example, under population contraction, all variants (deleterious and non-deleterious) are expected to become increasingly homozygous, but the proportion of deleterious and non-deleterious homozygous mutations would not change much in the short term.

Empirical studies suggest that most individuals within a species have similar levels of proportional genetic load. For example, van der Valk, et al. (*29*) evaluated load based on evolutionary conservation scores (GERP) across mammals, including resequencing data from multiple individuals, and found that intraspecific variability in genetic load is typically small (+/-SD 1.3%), and is smaller than interspecific variability. In a study of the vaquita (*Phocoena sinus*), intraspecific variation in the proportion of deleterious variants was also small relative to interspecies variability (*83*). Other studies also suggest that proportional drift load is not sensitive to recent demographic history (*84*). Although intraspecific variability can not be captured by sampling a single individual, these studies suggest that it will often provide a reasonable estimate of drift load that has accumulated over long evolutionary time periods in a given species.

The only two conspecific genomes in the dataset, the domestic dog and the village dog, have shared evolutionary histories until very recently, when lineages began to diverge in the Victorian Era. As expected, the domestic dog has slightly lower historical N_e (N_e=2,131) than the village dog (N_e=2,356), and the domestic dog had a slightly higher proportion of homozygous missense substitutions (0.3603) than the village dog (0.3591). These differences are very small compared to all of the species in the dataset, which ranged from 0.224-0.434 across 239 genomes, and the two dog genomes were 202nd and 206th when species were ranked by this metric. These measures of genetic load reflect older, shared evolutionary histories that have changed little with recent population divergence and different selective conditions. Other studies also suggest that proportional drift load is not sensitive to recent demographic history. In southern white rhinos, individuals sampled before a population bottleneck and after the bottleneck showed no difference in the proportions of homozygous missense mutations relative to homozygous synonymous mutations (*84*). In both of these examples, however, the populations

have diverged very recently (≲200 years). With increasing time since divergence between populations, samples from different populations are expected to become increasingly dissimilar.

Estimating heterozygosity and genetic load across homologous windows

We used the genomic distribution of heterozygosity and genetic load across mammalian taxa to train machine learning models for predicting conservation status (see below for machine learning methods). To generate matrices of heterozygosity and genetic load across homologous windows, we lifted over 50KB windows of 174 species to windows of the human genome using *halLiftover*, assigning the estimates of RoH, heterozygosity, mean phyloP across substitutions, and number of missense substitutions, from windows of each species to the window of the human genome. We averaged heterozygosity and the amount of RoH in each human-based window, and removed windows with fewer than 5KB that lifted over.

Statistical regression models of threatened status using genomic variables

We took three approaches to model conservation status across species using regression models. First, we used a phylogenetic logistic regression model, which accounts for evolutionary relationships across species. This model allowed us to test the significance of predictor variables, but does not readily make predictions for species with unknown threat status. Second, we used ordinal regression models, which estimate parameters based on specific IUCN categories. We included taxonomic order as a factor to account for phylogenetic relationships. These models allowed us to test the significance of predictor variables and make predictions for species with unknown threat status. Third, we used principal components (PCs) to summarize genomic variables, and tested the significance of PCs as predictors of threatened status using logistic regression. We also tested the ordinal regression and PC models within taxonomic orders to explore how the predictors of conservation status vary with taxonomy.

We incorporated genomic variables with taxonomic order and dietary trophic level, a known correlate of extinction risk (*65*), into these regression models. We subsetted the full dataset of 240 species to remove 16 domesticated species. We identified 13 possible predictor variables related to genomic heterozygosity and genetic load (table S2). We examined these numeric variables for normality by visualizing Q-Q plots, transforming as necessary to improve normality and rescaling all variables to a Z-score. We then removed the three species with an IUCN status of "Data Deficient".

We estimated model error by running the ordinal regression and PC regression models on 80% of the data and using the predict function from the R stats package to predict the threatened status of the remaining 20% of the data. Our estimate of model error was the mean of 100 runs with different data subsets.

We used a phylogenetic logistic regression model to determine which genomic features were most predictive of conservation status and to calculate the odds of a classification of "threatened" (IUCN NT, VU, EN, CR categories), as compared to non-threatened (IUCN LC category). We combined all four non-LC categories to increase the power of our analyses and balance sample sizes between threatened and non-threatened groups. Visual inspection of genomic variables suggested that the four threatened categories were more similar to one another than to the LC category (fig. S2). To select variables for inclusion into the final model, we used phyloglm from the R phylolm package (version 2.6.3)(*55*) with the phylogenetic tree generated from the X chromosome for the 240 species (*56*). We tested each of the 13 heterozygosity and load variables and two categorical covariates (diet category and wild versus captive status)

individually against threatened status, dropping variables that were not significant at a p=0.10 threshold. For the remaining numeric variables, we examined pairwise correlations and for pairs with a correlation greater than 0.7, we removed the variable of the pair with the higher p-value in the individual models predicting conservation status. We ran phyloglm for the final phylogenetic logistic regression model with the variables that remained after filtering for significance and correlation. We retained both categorical covariates (the diet category of the species and wild versus captive status of the short-read data sample) in the final model because they were significant (p<0.10) when considered individually, and to account for their possible influence on heterozygosity and fRoH estimates. We dropped four numeric variables that were not significant individually (p>0.10), and dropped three other numeric variables that were highly correlated with, but less significant than, another variable. The final model included the phylogenetic tree, two categorical covariates, three variables related to genome heterozygosity and fRoH, and three variables related to genetic load. In this final model, both diet category and wild versus captive status significantly predicted threatened status (p<0.05); and two numeric variables significantly predicted threatened status (p<0.05): harmonic mean of the historical effective population size and proportion of the genome in RoH. As expected, lower historical effective population sizes increase the odds of being classified as threatened. Contrary to expectations, a lower proportion of the genome in RoH increases the odds of being classified as threatened, likely due to the skewed distributions of RoH and the captive samples included in the analysis. None of the genetic load metrics were significant in this model.

We used an ordinal regression model to determine which genomic features were most predictive of IUCN category, to estimate the probability of each IUCN category, and to examine how these probabilities covary with taxonomic order, diet category, and wild versus captive status. Due to the sparsity of species in a number of taxonomic orders, for this model we used only five orders that had a sufficient number of species (Carnivora, Cetartiodactyla, Chiroptera, Primates, and Rodentia). To select variables for inclusion into the final model, we used polr from the R MASS package (version 7.3.51.4)(*85*). We tested each of the 13 heterozygosity and load variables and three categorical covariates individually against IUCN category, dropping variables that were not significant at a p=0.10 threshold. For the remaining numeric variables, we examined pairwise correlations and for pairs with a correlation greater than 0.7, we remove the variable of the pair with the higher p-value in the individual models. We dropped six numeric variables because they were not significant individually (p>0.10); we dropped three other variables due to high correlation with and lower significance than another variable. We ran polr for the final ordinal regression model with the variables that remained after significance and correlation filtering. The final model included all three covariates, one variable related to heterozygosity, and three variables related to genetic load, and had a cross validation (CV) error of 31% for classification of threatened versus non-threatened. In the final model, taxonomic order, diet category, and harmonic mean of the historical effective population size significantly (p<0.05) predicted IUCN status. As with the previous model, lower historical N_e indicated an increased probability of being classified as threatened. The impact of a lower historical N_e was greater on species with a diet classification of herbivores, as compared to omnivores and carnivores (Fig. 3B). We then modeled extinction risk within the three taxonomic orders with sufficient samples in threatened and non-threatened categories (Carnivora, Cetartiodactyla, and Primates), using the same process as above (excluding the taxonomic order variable) to select variables in order-specific ordinal regression models. When examining taxonomic order, the impact of historical N_e was reduced in Chiroptera and Rodentia (Fig. 3C).

To retain the information from all predictor variables and account for the correlation between them, we used principal component (PC) regression. Using the same 13 predictor variables, we removed species with missing data and ran a PC analysis using prcomp from the R stats package (version 3.6.1)(*86*). We ran a linear model to predict threatened status using lm from the R stats package, including the fewest PCs that cumulatively accounted for at least 80% of the variance in the data (table S3). For the three taxonomic orders with sufficient sample sizes, we used lm from the R stats package and the same set of PCs to run order-specific PC regression models. We tested the significance of the first five PCs, which accounted for greater than 80% of the cumulative variation in the predictor variables, in predicting threatened status. Two PCs were significant: PC1 (p=0.0038; explaining 35% of the total variance) and PC3 (p=5.6e-4; explaining 13% of the total variance). PC1 broadly represents heterozygosity and genetic load metrics and PC3 separates the mildly and severely deleterious mutations (table S3).

Given the importance of taxonomic order in all the models we examined, we tested the ordinal regression and PC models within the three taxonomic orders that had enough individuals in both non-threatened and threatened categories. Carnivora showed a complex relationship between threatened status and genomic variables. While a number of variables were significant when considered individually, none significantly impacted threatened status when considered together in the ordinal regression model. When PC regression was used to incorporate all the genomic variables while reducing dimensionality and correlation, the two most significant PCs were PC1 (p=0.07) and PC4 (p=0.04) that have major contributions from historical N_e and genetic load due to viable heterozygous loss of function (fig. S9). For Carnivora, the ordinal regression model had a CV error of 28% and the PC regression model had a CV error of 38%. Within Cetartiodactyla, there were no significant (p<0.05) predictors of threatened status in either the ordinal or PC regression models, however a few predictors were significant at a p=0.10 threshold, suggesting either a weak relationship and/or a lack of power due to small sample sizes. Additionally, Cetartiodactyla contains two groups with distinct ecological niches, one terrestrial and one aquatic, which may influence the genomic predictors of extinction risk in the two groups. The ordinal regression model had a CV error of 39% and the PC regression model had a CV error of 47%. Primates had a single variable that was significantly predictive of threatened status–the kurtosis of phylop, which is a measure of the tailedness of the distribution of phylop scores across substitutions. Primate species that are threatened tend to have fewer variants in the tail of the phylop distribution (phylolm, p=0.003), suggesting that purging of deleterious variants may be common in Primates (fig. S8). For Primates, the ordinal regression model had a CV error of 39% and the PC regression model had a CV error of 44%.

To make predictions for species classified as "Data Deficient", we used the predict function from the R stats package with the ordinal regression models and the PC regression models (excluding the phylogenetic PC regression model because there is no predict function available in the phylolm package).

<u>Machine learning (ML) methods for categorizing IUCN status using genomic features</u>

We next used random-forest based classification to identify the genomic features that predict “threatened” versus “non-threatened” status of species. We used two different genomic data types: 1) summary statistics of heterozygosity, RoH, and metrics of genetic load within homologous 50KB windows, and 2) genome-wide summary statistics related to heterozygosity, demographic history, and genetic load (table S4). For window-based summary statistics, we lifted over each genome to common coordinates of the human genome as described above (see

Estimating diversity across homologous windows). We generated five genomic feature matrices by estimating the following within windows of each genome: heterozygosity, RoH, mean phyloP of substitutions, number of missense substitutions, and number of missense substitutions at evolutionarily conserved sites. We ran the two genomic data types separately and combined, and additionally incorporated 39 numeric ecological features from the PanTHERIA database (table S4) to assess predictive performance of genomic features in comparison with ecological variables, considered a "gold standard" for prediction (*31*, *32*).

We started with five genomic feature window matrices in 57,509 homologous 50KB windows for at least 197 and up to 236 species depending on the statistic (table S5). Because we observed little impact of removing domesticated species in the regression models, we included them in the random forest models. We normalized counts of missense substitutions and counts of conserved missense substitutions by dividing by the total number of protein-coding variants for a given species. For each of the five genomic feature window matrices, we removed species that had missing values in more than 30,000 windows (which varied based on the statistic).

We included 13 genome-wide summary statistics describing demographic history, diversity, and genetic load (table S4). Of a total of 39 possible ecological features, the number of features included in each model depended on the number of species that had complete data for each model included in the training set, and this varied depending on which genomic feature dataset was used (window matrices, genome-wide summary statistics, or the two types combined).

We first split our dataset into a 75% train set and a 25% test set, and removed three data-deficient species (*Orcinus orca*, *Tragulus javanicus* and *Nannospalax galili*) lacking an IUCN status. This split was defined by a reproducible seed (ranging from 1 to 5), and repeated to test for robustness. Then, to prevent data leakage, we performed preprocessing and imputation steps using only the training data. We removed window-based metrics that had (1) missing values in more than 75% of the species, or (2) features with the mode value occuring in more than 75% of non-missing values. (All genome-wide summary statistics and ecological features passed these criteria.) Missing values were imputed within a feature vector using two methods (1) computing the median across all other species, or (2) by leveraging taxonomic order as follows: first, we compute the median of the species within the same genus; if there are no non-missing values, then we move up to compute the median value within the same family; then order. Missing values within the test set were imputed analogously using only the values within the training set.

We grouped the IUCN conservation status into two classes, threatened (NT/VU/EN/CR) and non-threatened (LC), with the goal of distinguishing between threatened and non-threatened species. Our models take as input the filtered window-based statistics, genome-wide summary statistics, and ecological variables. The output is a probability of the species being threatened.

Similar to previous work (*31*, *32*), we used random forest classifiers to assess the relationship between features and IUCN status. Random forests is an ensemble learning approach, making predictions by combining the outputs of hundreds of decision trees. We ran 5-fold cross validation on the training set to determine the optimal set of hyperparameters, which define the structure and learning process of the internal decision trees. Specifically, we tuned the number of decision trees, the maximum depth of the trees, and the number of features used at each decision to optimize a performance metric. For all models (except for the model trained on solely the 13 genomic summary variables), we added an additional hyperparameter governing the number of features selected during feature selection. During cross-validation, our median-based imputation was computed within each fold, whereas our phylogenetic imputation was computed on the entire training set. We used the area under the receiver operating characteristic

(AUROC) curve to evaluate performance. AUROC is a performance metric that estimates how well a model assigns (predicts) the correct output class, and is designed to be more robust to class imbalance in comparison to a metric such as accuracy. A model with AUROC of 0.5 has no predictive ability, whereas a model with AUROC of 1.0 has perfect predictive performance. Using the selected features and hyperparameters, we re-trained a model on the training set, evaluated it on the held-out test set, and reported the performance metric used during optimization. For all models, we ranked the features based on the model feature importance, a measure of the predictive power of the feature relative to other features in the model. Feature selection was performed in all cases except for genome summary, which had only 13 features. We used the scikit-learn 1.0.2 package for fitting all the models (*66*).

The number of species with values for ecological, genome-wide summary statistics, and window-based metrics varied, and so we ran two types of models: “individual” and “composite” models using 5-fold cross-validation for model selection. First we ran “individual” models including all species available for each dataset (table S5). The individual models help us evaluate the utility (i.e. predictive performance) of genomic variables as predictors of conservation status while leveraging all the data available for each metric. The method of imputation had an impact on predictive power, with imputation based on phylogeny showing superior performance over imputation based on median across all species regardless of taxonomic order (table S5). The model including only ecological variables across all available species had the best predictive AUROC (median across 5 training-test replicates was 0.88), while the models with genomic features had lower, but still good predictive power. Genomic window-based metrics varied in their predictive power, with AUROC ranging from 0.69-0.82 (table S5). The median AUROC values across five training-test replicates were 0.69 for the model with the proportion of missense substitutions, 0.70 for the proportion of conserved missense substitutions, 0.78 for RoH, 0.79 for heterozygosity, 0.74 for all window-based metrics combined, and 0.82 for the model with three window-based features combined (RoH, heterozygosity, and mean phyloP). The results suggest that windows of mean phyloP across substitutions, RoH, and heterozygosity were relatively more predictive than the other window-based features. Genome-wide summary variables were also somewhat predictive of threatened status, with a median AUROC of 0.68. There was little variance in model performance across the five training-test replicates for each individual model (mean coefficient of variation across models = 0.07, range=0.01-0.22; fig. S11).

To compare the effect of combining ecological and genomic variables on classification, we ran “composite” models, testing genomic (genome-wide summary and window-based) features and ecological features in the set of species for which both data were available (tables S4 and S6). We used the best performing window-based features derived from feature selection among the window-based features alone, and imputed missing data by phylogeny, which showed superior performance in the “individual” models. There was little variance in model performance across the five training-test replicates for each composite model (mean coefficient of variation across models = 0.08, range=0.03-0.21; fig. S12-S13). Models for 210 species with ecological variables and genomic summary statistics combined (median AUROC=0.85) modestly outperformed those with ecological variables alone (median AUROC=0.83). Among all 52 variables included in these models, there were three genomic variables that consistently appeared among the top 20 predictive features across replicates, 1) historical N_e (five replicates), 2) proportion of heterozygous missense variants in IMPC lethal genes (four replicates), and 3)

proportion of substitutions at conserved sites (three replicates) (fig. S14). Models including window-based features never outperformed models with ecological variables alone (table S6).

Our evaluation suggests that genomic variables provide reasonable predictive performance, demonstrating the utility of using genomic variables when ecological variables are unavailable. We note caveats to our models: the species included in the model affect the results, as AUROC differs between independent and composite models (tables S5 and S6); our sample size is small and including additional observations and species will likely improve predictions and decrease this stochasticity. Our study is a pilot that demonstrates the potential usefulness of genomic data for triaging data deficient species, and motivates further studies exploring larger datasets and with feature transformation (e.g. using principal components) for improved predictive performance.

<u>Predicting conservation status of Data Deficient species</u>

For the three species that are IUCN classified as "Data Deficient", we used both the regression and the random forest models to predict their probability of having a threatened status (Fig. 3D). The ordinal regression models generate predictions for each specific IUCN category; however, given the reduced power to distinguish between IUCN threatened categories due to small sample sizes in each, we focused on the broader classification of threatened versus non-threatened. From the regression models overall, the Upper Galilee Mountains blind mole rat (*Nannospalax galili*) is least likely to be a threatened species, with probabilities estimated at 12% and 14%. (Note there were not enough Rodentia species classified as threatened to do an order specific model). The Java lesser chevrotain (*Tragulus javanicus*) is also predicted to be a threatened species; however, probability estimates ranged from 27-63%. The higher risk prediction is from the within-order models. A killer whale (*Orcinus orca*) from the Norwegian herring-eating population (*87*), is likely to be in a threatened category, with probability estimates ranging from 62-68%.

Random forest model predictions for Data Deficient species differed somewhat from the regression-based predictions, but the relative likelihoods of threat for the three species were nonetheless consistent to the regression model predictions (Fig. 3D). All genomic feature-only models consistently predicted *Nannospalax galili* as the least likely to be threatened (median probability: 0.18, range: 0.11-0.44). *Tragulus javanicus* had a higher probability of being threatened, but was more likely to be classified as not threatened (median probability: 0.32, range: 0.24-0.49). *Orcinus orca* was the most likely to be threatened (median probability: 0.48, range: 0.35-0.61).

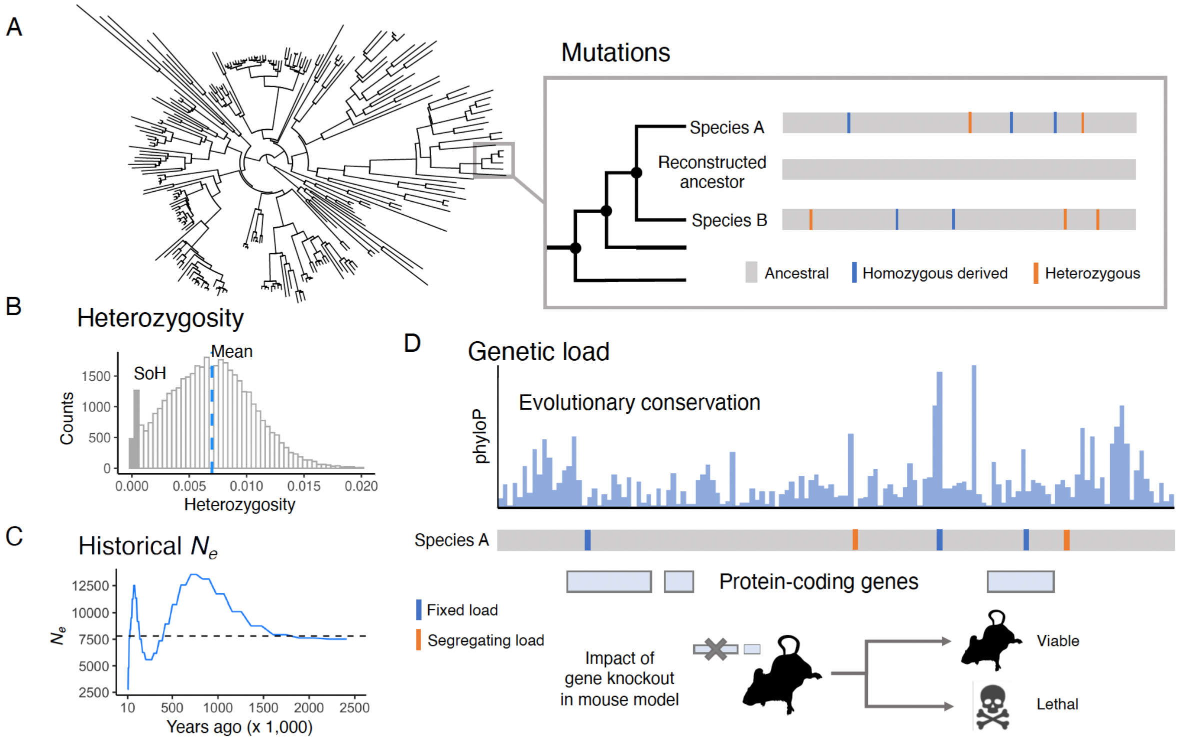

Figure S1.
Overview of methods for estimating heterozygosity, historical N_e, and genetic load across individual mammalian genomes. (A) For each species in the Zoonomia alignment, homozygous derived substitutions were estimated relative to the reconstructed sequence of the closest ancestral node in the phylogeny. Heterozygous variants were estimated from the short-read data mapped to the reference genome. **(B)** Mean heterozygosity and proportion of the genome in runs of homozygosity (fRoH) were estimated from the distribution of 50-kb genomic windows. **(C)** Historical effective population size (N_e) was estimated over time and summarized by the harmonic mean (dashed line). **(D)** Genetic load was inferred from the evolutionary conservation (measured by phyloP) of mutated positions, assuming that mutations at sites conserved across placental mammals are likely deleterious, and from the predicted impact of mutations in protein-coding genes, including single-copy genes with associated phenotypes in knockout mouse lines. Genetic load was estimated from the proportion of homozygous derived substitutions that were deleterious (fixed drift load), and the proportion of heterozygous variants that were deleterious (segregating mutational load), relative to total mutations of each type.

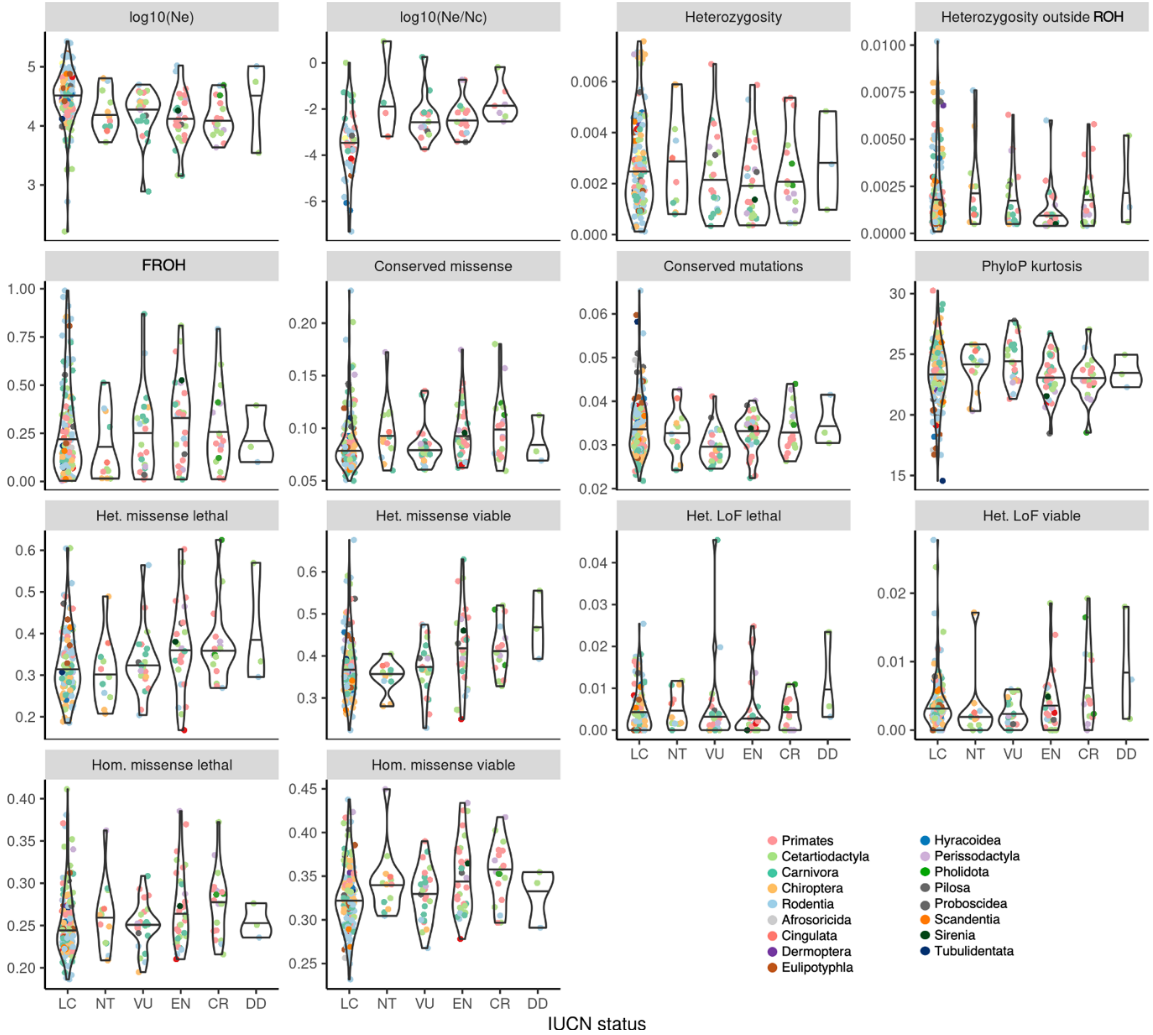

Figure S2.
Distribution of genomic variables of demography, diversity and genetic load in species across IUCN threat categories. All variables (except N_e/N_c) were used in regression and machine learning models to predict threatened and non-threatened IUCN status (see tables S2 and S4).

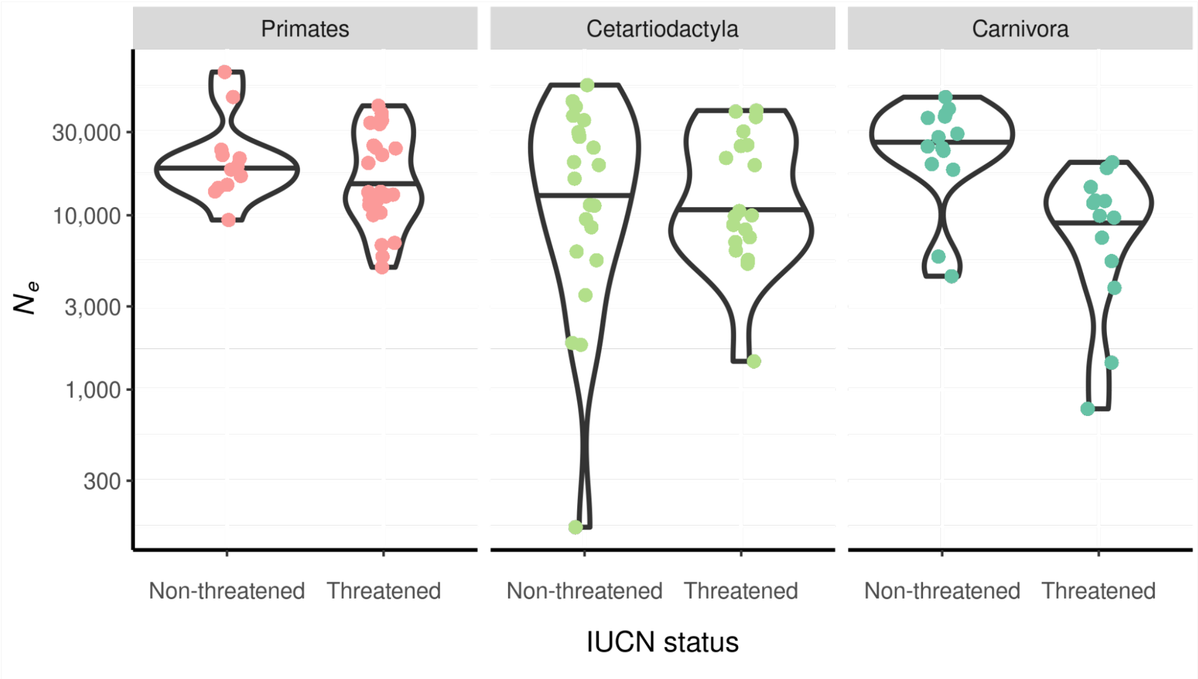

Figure S3.
Effective population sizes (N_e) were significantly smaller in threatened compared to non-threatened species within two of three taxonomic orders with enough samples in both threat categories to test: Cetartiodactyla (phylolm, p=0.023) and Carnivora (p=2.4e-5), but not Primates (phylolm, p=0.31).

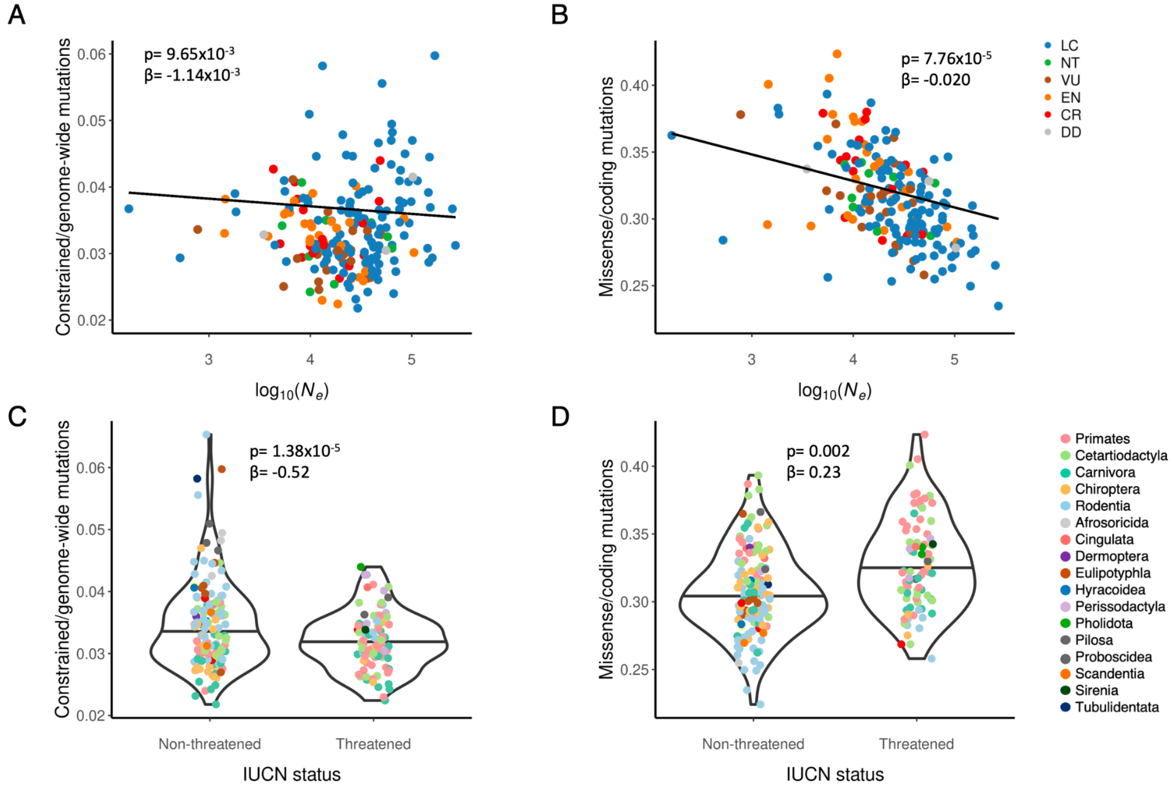

Figure S4.

Contrasting patterns of drift load based on conserved sites across the genome and missense substitutions in genes. A) N_e was negatively correlated with the proportion of homozygous substitutions that were at evolutionarily conserved sites (phylolm, p=9.6e-3, β=-0.0011, where a 10-fold increase in N_e corresponds to a decrease in proportion of 0.0011). B) N_e was negatively correlated with the proportion of homozygous missense substitutions that were at evolutionarily conserved sites (phylolm, p=7.76e-5, β= -0.020, where a 10-fold increase in N_e corresponds to a decrease in proportion of 0.020). Lines show coefficients estimated with phylogenetic correction using phylolm. C) Species that had proportionally fewer homozygous substitutions at evolutionarily conserved sites across the genome were more likely to be threatened (phyloglm; p=1.38e-05; β= -0.52). D) Species that had proportionally more homozygous missense substitutions in protein-coding genes were more likely to be threatened (phyloglm; p=0.002; β= 0.23).

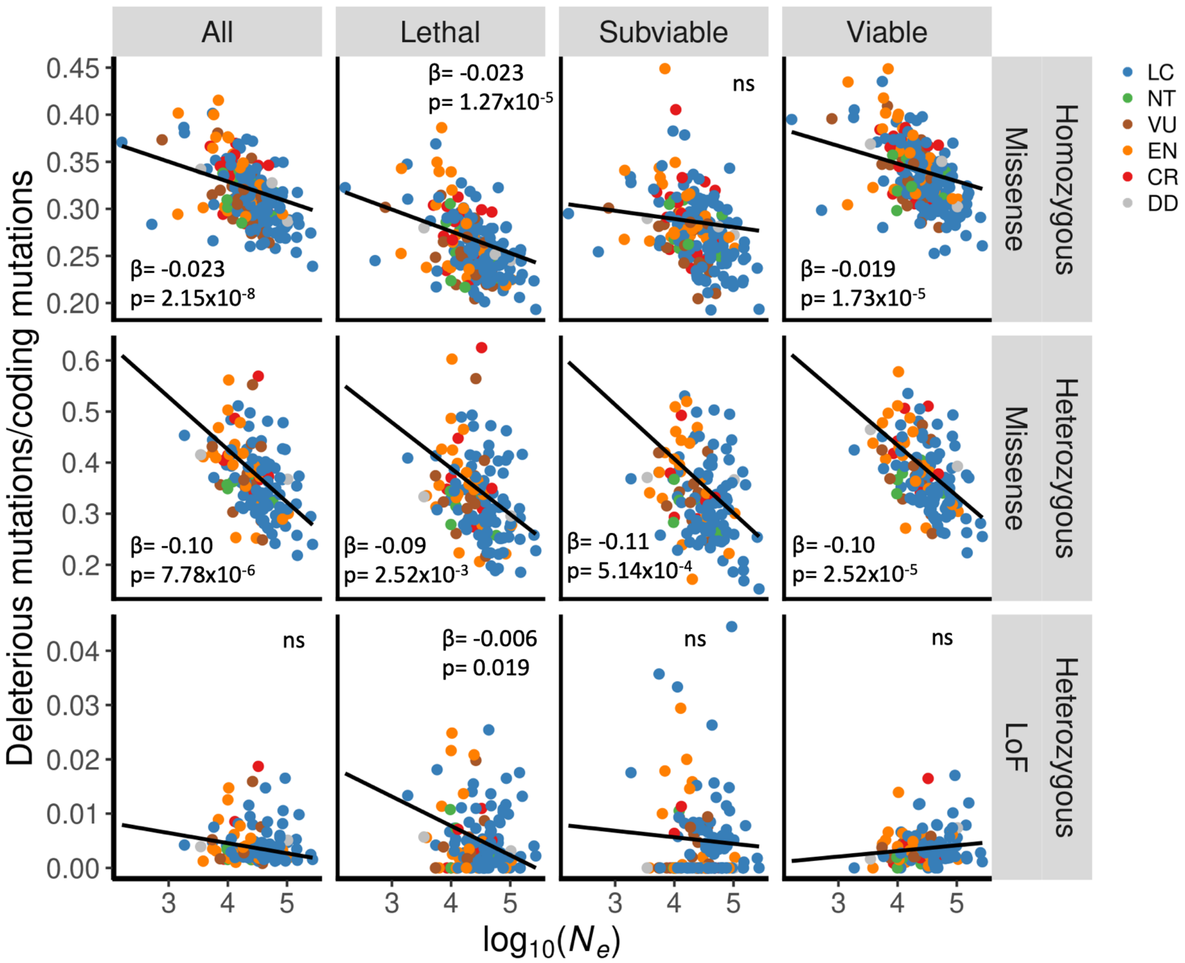

Figure S5.
Proportion of heterozygous loss of function (LoF), heterozygous missense, and homozygous missense mutations in protein-coding genes classified as lethal, subviable or viable in knockout mice as a function of harmonic mean N_e across species. For most IMPC categories, proportions of heterozygous and homozygous missense alleles were negatively correlated with harmonic mean of N_e, but heterozygous LoF alleles were generally not. P-values and coefficients were estimated using phylogenetic correction in phylolm.

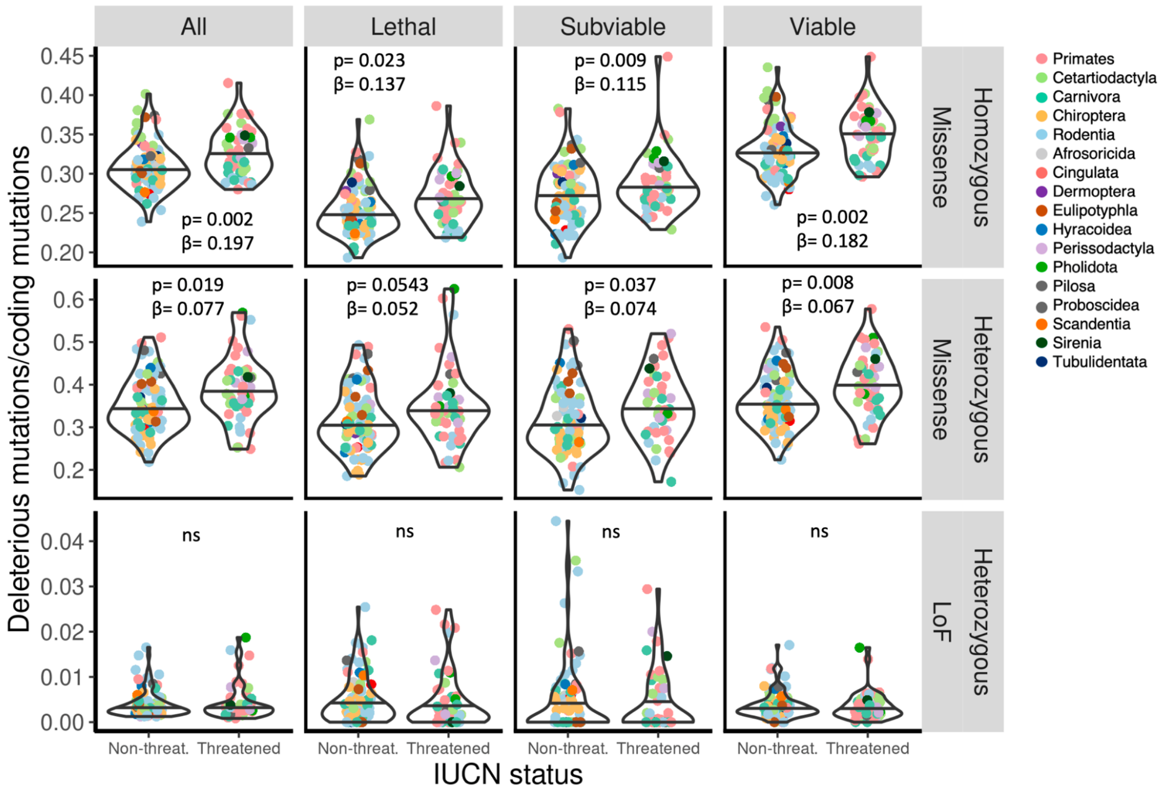

Figure S6.

Proportion of heterozygous LoF, heterozygous missense, and homozygous missense mutations in protein-coding genes classified as lethal, subviable or viable in knockout mice for non-threatened and threatened species. For most IMPC categories, species with proportionally more heterozygous and homozygous missense alleles were more likely to be threatened. P-values shown were estimated using phylogenetic correction in phyloglm. β coefficients indicate the change in odds of being threatened with a 1% increase in deleterious mutations. Phylogenetically corrected means did not significantly differ (all p>0.15).

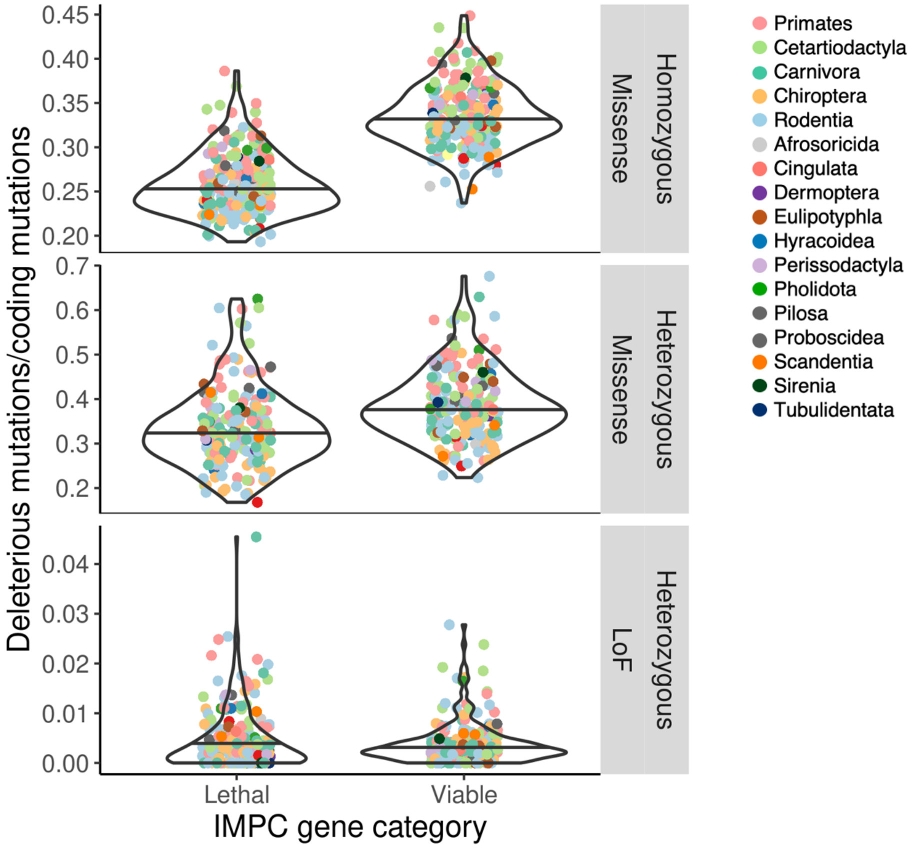

Figure S7.

Proportionally fewer missense mutations in genes associated with lethal phenotypes. Missense mutations were less frequent in genes classified as IMPC lethal relative to genes classified as IMPC viable (ANOVA, $p<2e-16$ and $p=4.42e-9$ for homozygous and heterozygous mutations, respectively). The difference between the two categories for heterozygous LoF alleles was not significant ($p=0.19$).

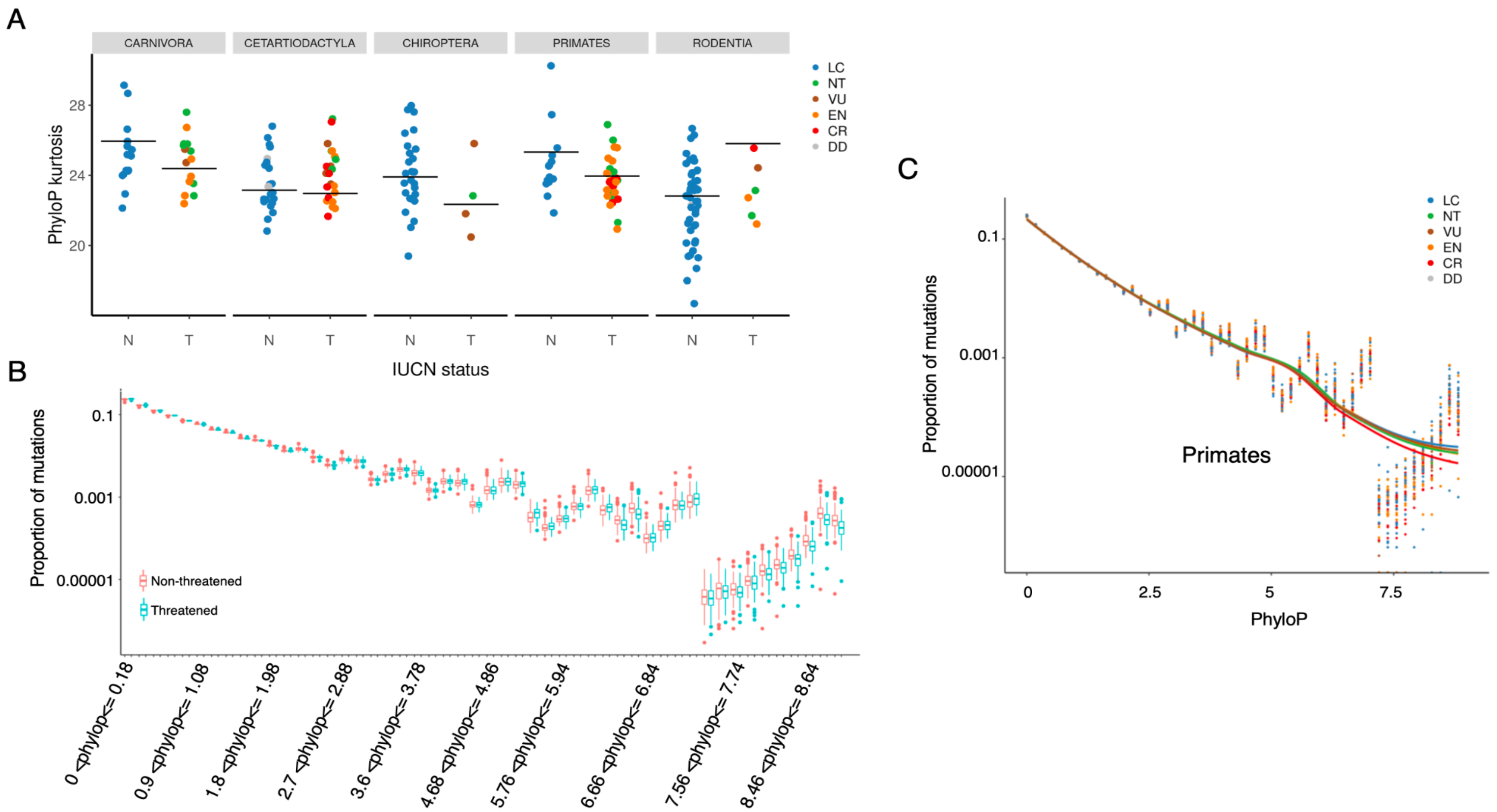

Figure S8.
Genome-wide conservation scores suggest fewer substitutions at highly conserved sites in threatened species. A) Kurtosis of the phyloP distribution across substitutions (larger numbers reflect fatter right tails) was significantly lower in threatened species across all taxa (p=0.004), and within Primates (p=0.003) and Carnivora (p=0.019). Horizontal lines show coefficients of means after phylogenetic correction in phylolm. B) Proportion of genome-wide substitutions in phyloP bins for threatened and non-threatened species, showing fewer substitutions in higher phyloP bins in threatened compared to non-threatened species across species. (C) Proportion of genome-wide substitutions in phyloP bins for threatened and non-threatened species, showing fewer substitutions in higher phyloP bins in threatened compared to non-threatened species within Primates. Lines show smoothed means for IUCN categories.

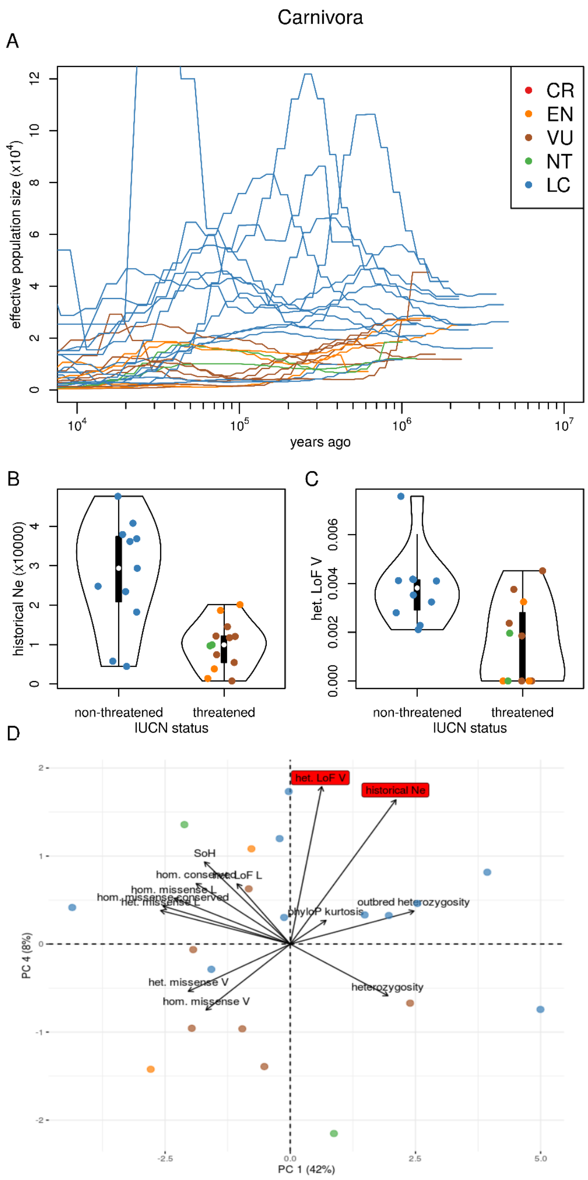

Figure S9.

Predictive metrics for Carnivora A) Plots of N_e through time estimated from PSMC and used to calculate historical N_e. B) Historical N_e is significantly lower in threatened species (ordinal regression, p=0.0055). C) The proportion of heterozygous variants that were loss of function in genes categorized as viable (het. LOF V) is significantly lower in threatened species (ordinal regression, p=0.0064). D) PCA space of the most significant PCs (PC1, p=0.075; PC4, p=0.040) of genomic predictor variables in PC regression models. Vectors indicate variable loading with the two most significant variables from ordinal regression models shown in red. Dots represent species and show their scores in PCA space. Colors in all panels represent the IUCN category of the species. SoH=runs of homozygosity.

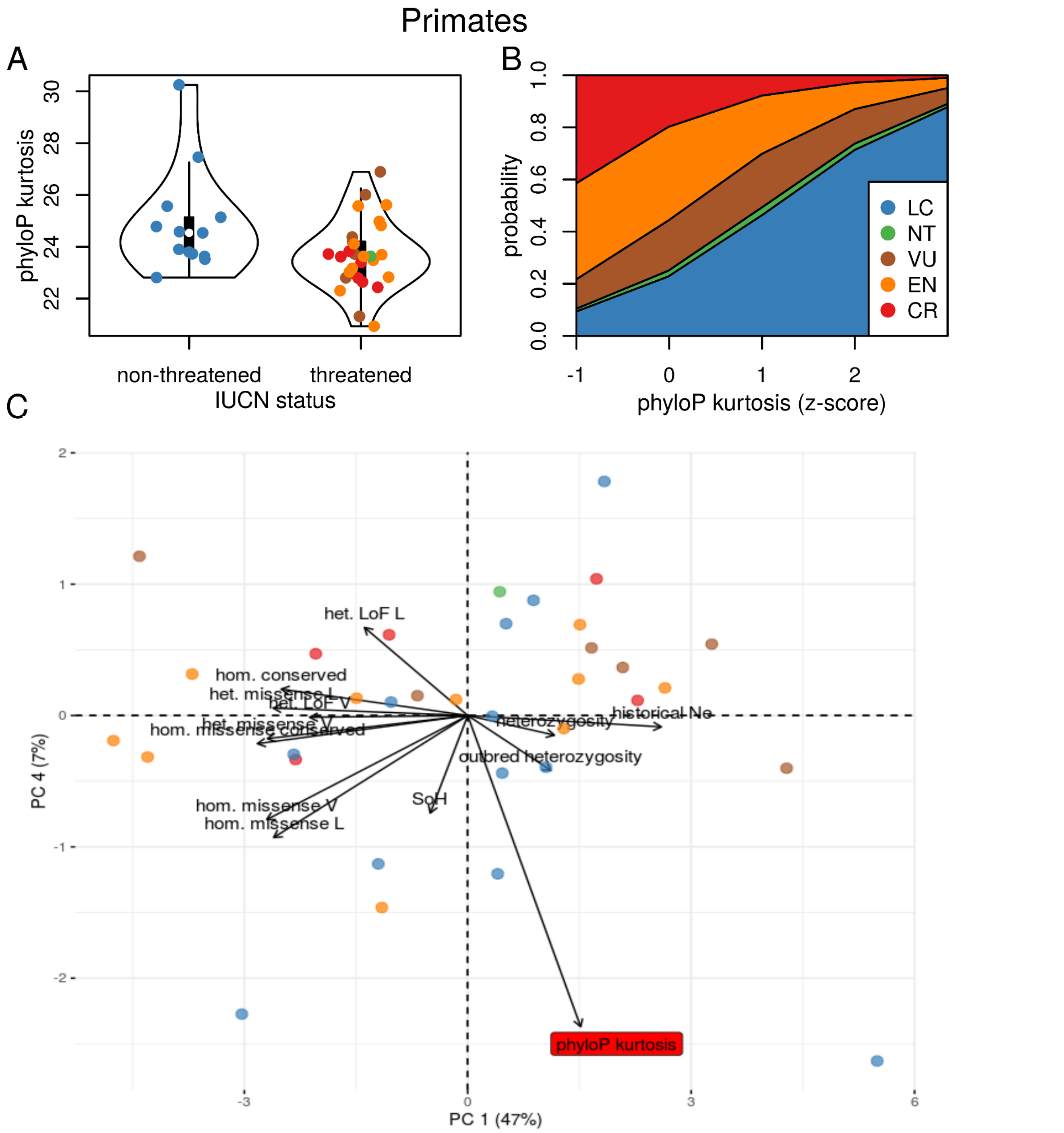

Figure S10.

Predictive metrics for Primates A) PhyloP kurtosis is significantly lower in threatened species (phylolm, p=0.003). B) Probability of IUCN categories for phyloP kurtosis score in the ordinal regression model (p=0.01). C) PCA space of genomic predictor variables showing PC1 (p=0.62) and the most significant predictor in the PC regression model PC4 (p=0.063). Vectors indicate variable loading, with PhyloP kurtosis (shown in red) as the major contributor to PC4. Dots represent species and show their scores in PCA space. Colors in all panels represent the IUCN category of the species. SoH=runs of homozygosity.

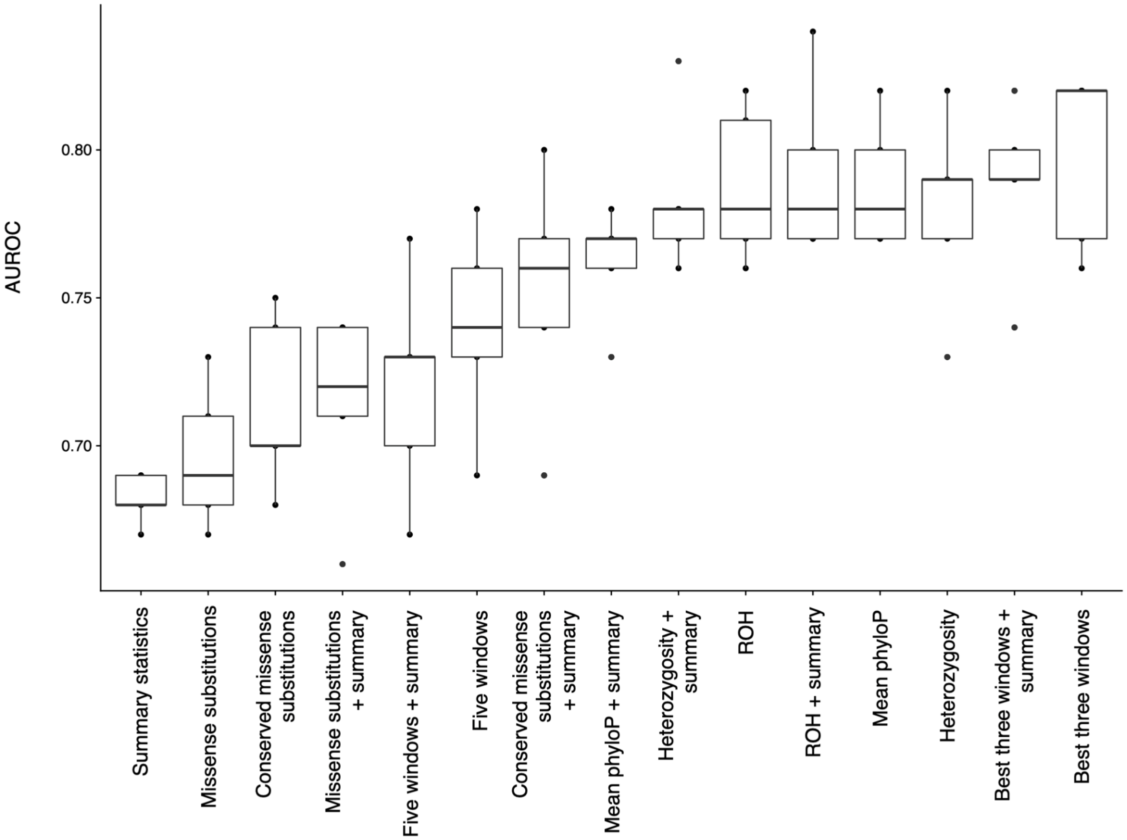

Figure S11.
Performance measurements (AUROC) across five training-test replicates of "individual" models that included window-based metrics and/or genome-wide summary statistics (table S5; see table S4 for data descriptions).

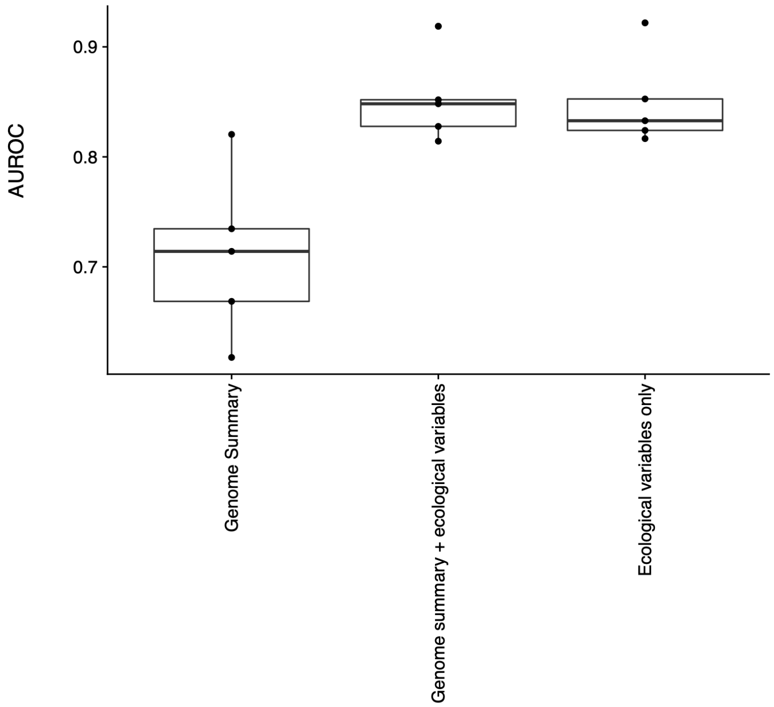

Figure S12.
Performance measurements (AUROC) across five training-test replicates of “composite” models that included genome-wide summary statistics, ecological variables, or both (table S6; see table S4 for descriptions).

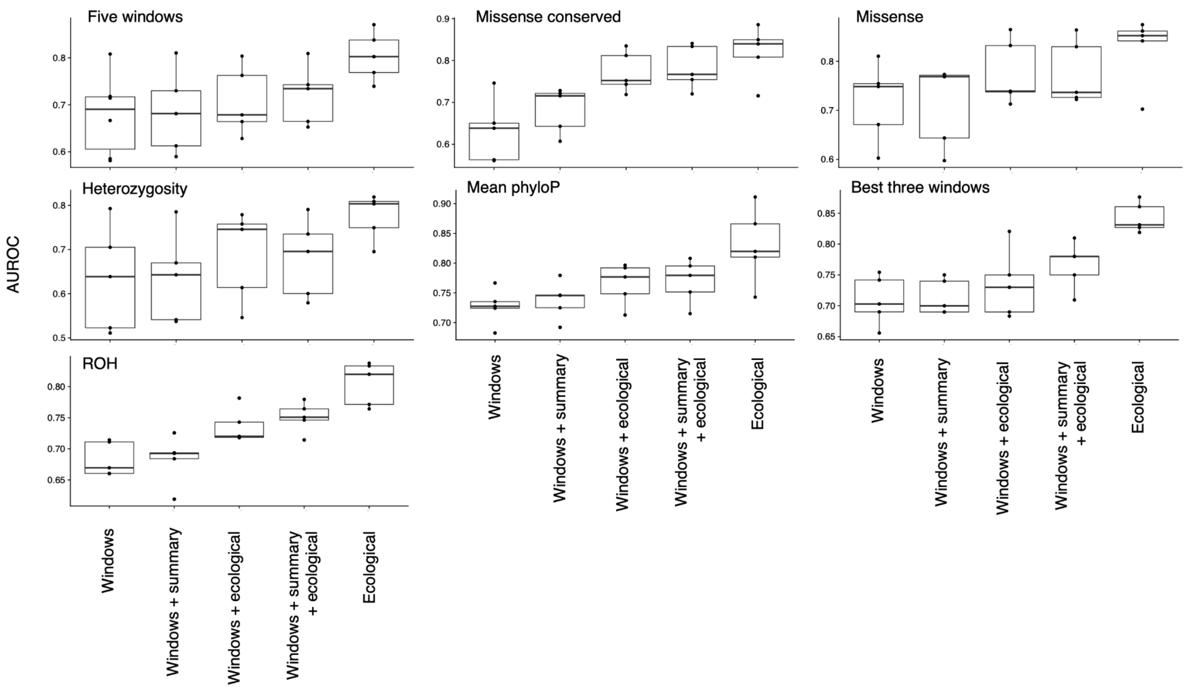

Figure S13.
Performance measurements (AUROC) across five training-test replicates of "composite" models that included genomic window-based metrics, genome-wide summary statistics, ecological variables, or combinations of these data types (table S6; see table S4 for descriptions).

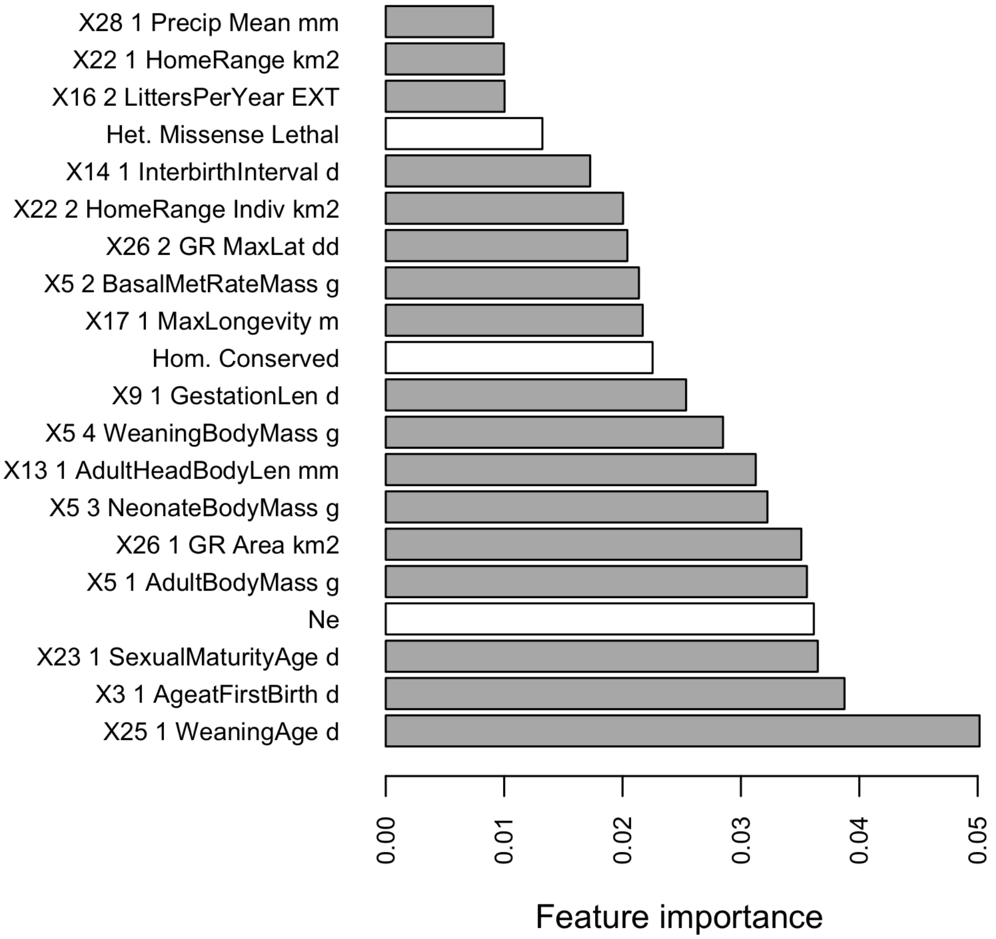

Figure S14.
Top 20 most important features in random forest models to predict conservation status. Most important features from the model including 13 genomic summary variables and 40 ecological variables across (AUROC=0.85). Feature importance was averaged across five test-training replicates. Genomic features are highlighted in white and ecological features from PanTHERIA are in dark gray.

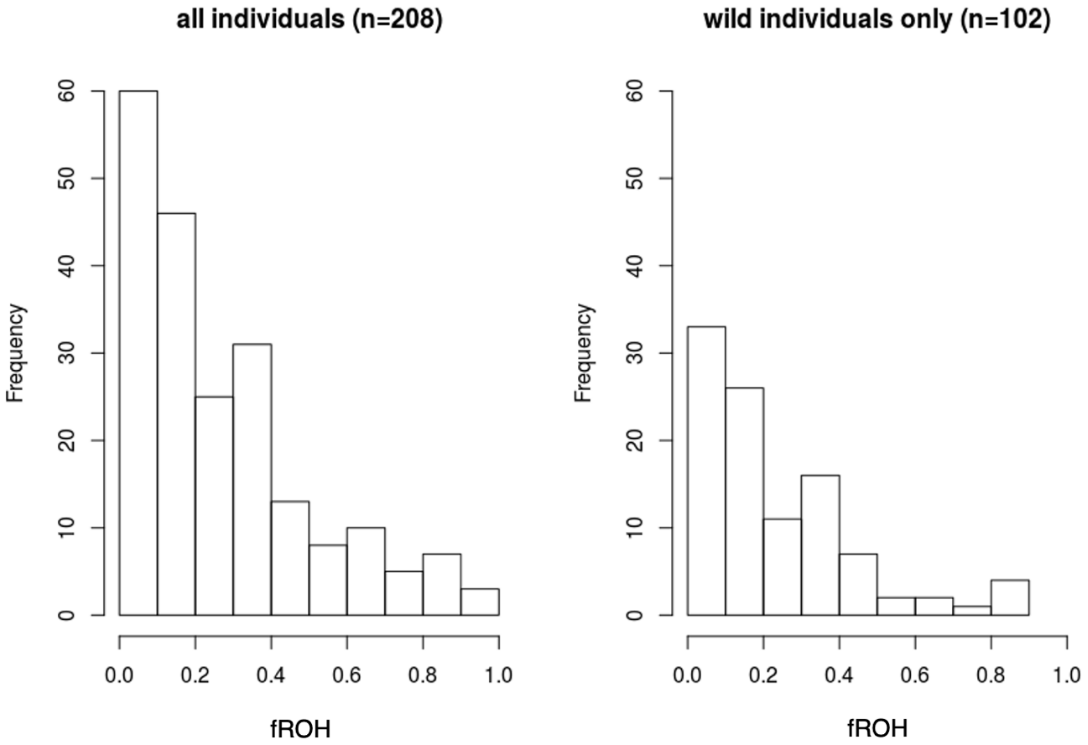

Figure S15.
Distribution of fRoH values for A) all species with fRoH estimates and B) species represented by a wild caught individual only.

Table S1. Data for the 241 Zoonomia species analyzed, including species name, taxonomic order, diet classification, wild or captive status of the sequenced sample, IUCN Red List category, NCBI accession numbers, genome contiguity statistic (contig N50), generation time, census size (Nc), and values for 13 genomic summary statistics used in analyses.

Table S2. Details and results for variables used in statistical regression models, including the transformation used to increase normality of the distribution and number of samples with an estimate. Model results indicate if the variable was dropped from the final model (and the reason it was dropped) or the p-values and untransformed coefficients for the final model. Bold values indicate the variable was significant in the model at the p=0.05 threshold.

**variable name**	**variable description**	**transform**	**sample size**	**logistic regression**	**ordinal regression**	**Carnivora ordinal regression**	**Cetartiodactyla ordinal regression**	**Primate ordinal regression**
historical N_e	harmonic mean of temporal estimates of N_e from PSMC	log	210	**p=3.8e-05, coefficient=-1.42**	**p=0.009, coefficient=-0.85**	p=0.29, coefficient=-1.17	dropped (p=0.40)	dropped (p=0.14)
heterozygosity	genome-wide heterozygosity	none	209	dropped (correlated)	dropped (p=0.18)	dropped (p=0.22)	dropped (p=0.25)	dropped (p=0.32)
outbred heterozygosity	mode of heterozygosity in non-RoH regions	log	209	p=0.25, coefficient=-0.33	dropped (p=0.17)	p=0.68, coefficient=0.52	dropped (p=0.33)	dropped (p=0.69)
fRoH	percentage of the genome in RoH	square root	208	**p=0.028, coefficient=-0.63**	dropped (p=0.97)	dropped (p=0.83)	p=0.084, coefficient=0.88	dropped (p=0.56)
hom. conserved	proportion of homozygous substitutions with phylop>2.27	log	241	p=0.19, coefficient=-0.31	p=0.99, coefficient=-0.0055	dropped (p=0.31)	dropped (p=0.27)	dropped (p=0.80)
phyloP kurtosis	tailedness of phylop distribution across substitutions	none	241	dropped (p=0.33)	dropped (p=0.33)	dropped (p=0.47)	dropped (p=0.70)	**p=0.023, coefficient=-1.06**
hom. missense conserved	proportion of hom. missense that are at sites with phylop>2.27	none	239	dropped (correlated)	dropped (correlated)	dropped (p=0.30)	dropped (p=0.47)	dropped (p=0.38)
het. missense L	in lethal genes, the proportion of heterozygous coding variants that are missense	log	190	dropped (p=0.12)	dropped (correlated)	p=0.18, coefficient=2.23	dropped (p=0.76)	dropped (p=0.98)
het. missense V	in viable genes, the proportion of heterozygous coding variants that are missense	log	196	dropped (p=0.16)	p=0.40, coefficient=-0.23	p=0.99, coefficient=0.0086	dropped (p=0.28)	dropped (p=0.59)
het. LoF L	in lethal genes, the proportion of heterozygous coding variants that are LoF	log (+0.01)	190	p=0.41, coefficient=-0.15	dropped (p=0.56)	dropped (p=0.81)	p=0.60, coefficient=-0.29	dropped (p=0.55)
het. LoF V	in viable genes, the proportion of heterozygous coding variants	log (+0.001)	196	dropped (p=0.62)	dropped (p=0.77)	p=0.067, coefficient=-1.15	dropped (p=0.32)	dropped (p=0.14)

	that are LoF							
hom. missense L	in lethal genes, the proportion of homozygous coding variants that are missense	log	239	dropped (correlated)	dropped (correlated)	dropped (p=0.65)	dropped (p=0.37)	dropped (p=0.53)
hom. missense V	in viable genes, the proportion of homozygous coding variants that are missense	none	239	p=0.38, coefficient=0.25	p=0.59, coefficient=-0.17	dropped (p=0.55)	dropped (p=0.26)	dropped (p=0.28)
order	taxonomic order	NA	241	NA	**p=0.023, 0.045,** 0.41, 0.93; **coefficients=-2.12, -1.67,** 0.62, 0.058	NA	NA	NA
diet	diet type (herbivore, omnivore, carnivore)	NA	241	**p=0.014**, 0.64; **$coefficient_h$=1.46**, $coefficient_o$=0.28	**p=0.0037,** 0.86; **$coefficient_h$=1.88**, $coefficient_o$=0.11	dropped (rank-deficient)	p=0.15, **0.034**; $coefficient_h$=1.59, **$coefficient_o$=4.07**	dropped (rank-deficient)
wild	sample from wild or captive population	NA	225	**p=0.020, coefficient=-0.97**	p=0.96, coefficient=-0.20	dropped (p=0.34)	p=0.93, coefficient=0.099	dropped (p=0.30)

Table S3. Loadings for genomics summary statistics of the first five principal components (PCs; accounting for >80% of total variance) used in models to predict threatened status of species across all orders. P-values are reported for PCs that significantly predicted threatened status. Summary statistics are described in table S2.

	PC1 (p=0.0038)	PC2	PC3 (p=5.6e-4)	PC4	PC5
hom. conserved	0.215	0.305	-0.302	0.403	-0.080
phyloP kurtosis	-0.122	-0.282	0.317	-0.483	0.171
historical N_e	-0.306	0.091	-0.384	0.191	0.125
heterozygosity	-0.242	0.419	-0.118	-0.244	-0.056
outbred heterozygosity	-0.229	0.416	-0.004	-0.273	0.023
fRoH	0.124	-0.455	-0.082	0.322	-0.027
hom. missense conserved	0.386	0.283	0.096	0.104	0.039
het. missense L	0.347	-0.167	-0.258	-0.24	-0.066
het. missense V	0.375	-0.148	-0.112	-0.216	0.048
het. LoF L	0.111	-0.044	-0.431	-0.392	-0.644
het. LoF V	0.107	-0.025	-0.517	-0.221	0.713
hom. missense L	0.384	0.264	0.177	-0.062	0.047
hom. missense V	0.371	0.248	0.26	-0.089	0.103

Table S4. Genomic and ecological variables used in machine learning models to predict IUCN status in mammalian species. Genomic window-based variables were estimated within 50KB homologous windows lifted over to the human genome. Genomic summary variables are genome-wide summary statistics. Ecological variables were obtained from the PanTHERIA database.

	Genomic window-based variables	***Description***
1	Heterozygosity	Mean heterozygosity in homologous 50KB windows
2	RoH	Mean RoH in homologous 50KB windows
3	Mean phyloP	Mean phyloP across substitutions in homologous 50KB windows
4	Missense conserved substitutions	Proportion of homozygous missense substitutions in homologous 50KB windows that are at evolutionarily conserved sites (phyloP>2.27)
5	Missense substitutions	Proportion of homozygous coding substitutions in homologous 50KB windows that are missense
	Genomic summary variables	
1	Historical N_e	Harmonic mean of historical effective population size
2	Heterozygosity	Mean genome-wide heterozygosity
3	Heterozygosity (non-RoH)	Mean heterozygosity outside of RoH
4	fRoH	Proportion of the genome in RoH
5	Conserved homozygous	Proportion of homozygous that are at evolutionarily conserved sites (phyloP>2.27)
6	PhyloP kurtosis	Kurtosis of phyloP across homozygous
7	Missense homozygous at conserved sites	Proportion of missense homozygous that are at evolutionarily conserved sites (phyloP>2.27)
8	Heterozygous missense lethal	Proportion of heterozygous coding variants in IMPC lethal genes that are missense
9	Heterozygous missense viable	Proportion of heterozygous coding variants in IMPC viable genes that are missense
10	Heterozygous LoF lethal	Proportion of heterozygous coding variants in IMPC lethal genes that are LoF
11	Heterozygous LoF viable	Proportion of heterozygous coding variants in IMPC viable genes that are LoF
12	Homozygous missense lethal	Proportion of homozygous coding variants in IMPC lethal genes that are missense
13	Homozygous missense viable	Proportion of homozygous coding variants in IMPC viable genes that are missense
	Ecological variables	
1	X5 1 Adult Body Mass g	Mass of adult (or age unspecified) live or freshly-killed specimens (excluding pregnant females) using captive, wild, provisioned, or unspecified populations; male,

		female, or sex unspecified individuals; primary, secondary, or extrapolated sources; all measures of central tendency; in all localities
2	X13 1 Adult Head BodyLen mm	Total length from tip of nose to anus or base of tail of adult (or age unspecified) live, freshly-killed, or museum specimens using captive, wild, provisioned, or unspecified populations; male, female, or sex unspecified individuals; primary, secondary, or extrapolated sources; all measures of central tendency; in all localities
3	X2 1 Age at Eye Opening d	Age at which both eyes are fully open after birth using captive, wild, provisioned, or unspecified populations; male, female, or sex unspecified individuals; primary, secondary, or extrapolated sources; all measures of central tendency; in all localities
4	X3 1 Age at First Birth d	Age at which females give birth to their first litter (eutherians), or their young attach to teats (metatherians) or hatch out (monotremes), using non-captive, wild, provisioned, or unspecified populations; primary, secondary, or extrapolated sources; all measures of central tendency; in all localities
5	X18 1 Basal Met Rate mL O2 hr	Basal metabolic rate of adult (or age unspecified) individual(s) using captive, wild, provisioned, or unspecified populations; male, female, or sex unspecified individuals; primary, secondary, or extrapolated sources; all measures of central tendency; in all localities. Metabolic rate was measured when individual(s) were experiencing neither heat nor cold stress (i.e. are in their thermoneutral zone); are resting and calm; and are post–absorptive (are not digesting or absorbing a meal) and data were only accepted where there was also a measure of body mass for the same individual(s)
6	X5 2 Basal Met Rate Mass g	Mass of individual(s) from which the basal metabolic rate was taken
7	X6 1 Diet Breadth	Number of dietary categories eaten by each species measured using any qualitative or quantitative dietary measure, over any period of time, using any assessment method, for non-captive or non-provisioned populations; adult or age unspecified individuals, male, female, or sex unspecified individuals; primary, secondary, or extrapolated sources; all measures of central tendency; in all localities. Categories were defined as vertebrate, invertebrate, fruit, flowers/nectar/pollen, leaves/branches/bark, seeds, grass and roots/tubers
8	X9 1 Gestation Len d	Length of time of non-inactive fetal growth, using captive, wild, provisioned, or unspecified populations; male, female, or sex unspecified individuals; primary, secondary, or extrapolated sources; all measures of central tendency; in all localities. Gestation was measured between specified start and end points as follows: Start points – conception, fertilization, first observed copulation, fertilization, implantation, laying, palpably pregnant, removal of pouch young, capture (except marsupials) or unspecified. End points – birth, hatching or unspecified
9	X12 1 Habitat Breadth	Number of habitat layers used by each species measured using any qualitative or quantitative time measure, for non-captive populations; adult or age unspecified individuals, male, female, or sex unspecified individuals; primary, secondary, or extrapolated sources; all measures of central tendency; in all localities. Categories were defined as above ground dwelling, aquatic, fossorial and ground dwelling
10	X22 1 Home Range km2	Size of the area within which everyday activities of individuals or groups (of any type) are typically restricted, estimated by either direct observation, radio telemetry, trapping or unspecified methods over any duration of time, using non-captive populations; male, female, or sex unspecified individuals; primary, secondary, or extrapolated sources; all measures of central tendency; in all localities
11	X22 2 Home Range Indiv km2	Size of the area within which everyday activities of individuals are typically restricted, estimated by either direct observation, radio telemetry, trapping or unspecified methods over any duration of time, using non-captive populations; male, female, or sex unspecified individuals; primary, secondary, or extrapolated sources; all measures of central tendency; in all localities
12	X14 1 Inter birth Interval d	The length of time between successive births of the same female(s) after a successful or unspecified litter using non-captive, wild, provisioned, or unspecified populations; primary, secondary, or extrapolated sources; all measures of central tendency; in all

		localities
13	X15 1 Litter Size	Number of offspring born per litter per female, either counted before birth, at birth or after birth, using captive, wild, provisioned, or unspecified populations; male, female, or sex unspecified individuals; primary, secondary, or extrapolated sources; all measures of central tendency; in all localities
14	X16 1 Litters Per Year	Number of litters per female per year using non-captive, wild, provisioned, or unspecified populations; male, female, or sex unspecified individuals; primary, secondary, or extrapolated sources; all measures of central tendency; in all localities
15	X17 1 Max Longevity m	Maximum adult age measured either through direct observation, capture-recapture estimates, projected from physical wear or unspecified, using captive, wild, provisioned, or unspecified populations; male, female, or sex unspecified individuals; primary, secondary, or extrapolated sources; in all localities
16	X5 3 Neonate Body Mass g	Mass of live or freshly-killed specimens of infants at either a near term embryonic stage, birth, immediately after birth or up to an age of seven days after birth, using captive, wild, provisioned, or unspecified populations; male, female, or sex unspecified individuals; primary, secondary, or extrapolated sources; all measures of central tendency; in all localities
17	X21 1 Population Density n km2	Number of individuals per square kilometer, estimated with either direct, indirect or unspecified counts, measured in any area size within a human, ecological or unspecified boundary, over any duration of time, using non-captive, non-provisioned populations; male, female, or sex unspecified individuals; primary, secondary, or extrapolated sources; all measures of central tendency; in all localities
18	X10 1 Population Grp Size	Number of individuals, adults or definition unspecified in a group that spends the majority of their time in a 24 hour cycle together, measured over any duration of time, using non-captive populations; male, female, or sex unspecified individuals; primary, secondary, or extrapolated sources; all measures of central tendency; in all localities
19	X23 1 Sexual Maturity Age d	Age when individuals are first physically capable of reproducing, defined as either physically sexually mature, age at first mating or unspecified (males and females), age at first estrus or age at first pregnancy (females only), age at spermatogenesis or age at testes descent (males only), using captive, wild, provisioned, or unspecified populations; male, female, or sex unspecified individuals, primary, secondary, or extrapolated sources; all measures of central tendency; in all localities
20	X10 2 Social Grp Size	Number of individuals, adults or definition unspecified in a group that spends the majority of their time in a 24 hour cycle together where there is some indication that these individuals form a social cohesive unit, measured over any duration of time, using non-captive populations; male, female, or sex unspecified individuals; primary, secondary, or extrapolated sources; all measures of central tendency; in all localities
21	X24 1 Teat Number	Total number of teats present, using captive, wild, provisioned, or unspecified populations; male, female, or sex unspecified individuals; primary, secondary, or extrapolated sources; all measures of central tendency; in all localities
22	X25 1 Weaning Age d	Age when primary nutritional dependency on the mother ends and independent foraging begins to make a major contribution to the offspring's energy requirements, measured as either weaning/lactation length, nutritionally independent, first solid food, last observed nursing, age at first flight (bats only), age at pouch exit or length of teat Attachment (marsupials only) or unspecified definition, using captive, wild, provisioned, or unspecified populations; male, female, or sex unspecified individuals; primary, secondary, or extrapolated sources; all measures of central tendency; in all localities
23	X5 4 Weaning Body Mass g	Mass of live or freshly-killed specimens of weanlings, using captive, wild, provisioned, or unspecified populations; male, female, or sex unspecified individuals; primary, secondary, or extrapolated sources; all measures of central tendency; in all localities

24	X16 2 Litters Per Year EXT	Species medians of the consolidated values
25	X26 1 GR Area km2	Total extent of a species range with a global equal-area projection
26	X26 2 GR Max Lat dd	Maximum latitudinal extent of each species range calculated using a global geographic projection (decimal degrees)
27	X26 3 GR Min Lat dd	Minimum latitudinal extent of each species range calculated using a global geographic projection (decimal degrees)
28	X26 4 GR Mid Range Lat dd	Median latitudinal extent of each species range calculated using a global geographic projection (decimal degrees)
29	X26 5 GR Max Long dd	Maximum longitudinal extent of each species range calculated using a global geographic projection (decimal degrees)
30	X26 6 GR Min Long dd	Minimum longitudinal extent of each species range calculated using a global geographic projection (decimal degrees)
31	X26 7 GR Mid Range Long dd	Median longitudinal extent of each species range calculated using a global geographic projection (decimal degrees)
32	X27 1 Hu Pop Den Min n km2	Minimum human population density (persons per km^2) using the Gridded Population of the World (GPW) (CIESIN and CIAT 2005) for 1995
33	X27 2 Hu Pop Den Mean n km2	Mean human population density (persons per km^2) using the Gridded Population of the World (GPW) (CIESIN and CIAT 2005) for 1995
34	X27 3 Hu Pop Den 5p n km2	5th percentile human population density (persons per km^2) using the Gridded Population of the World (GPW) (CIESIN and CIAT 2005) for 1995
35	X27 4 Hu Pop Den Change	Mean rate of increase in human population density using the Gridded Population of the World (GPW) (CIESIN and CIAT 2005) for 1990 and 1995 as: (1995–1990)/1990
36	X28 1 Precip Mean mm	Mean monthly precipitation (mm) calculated using data from ftp://ftp.ngdc.noaa.gov/Solid_Earth/Ecosystems/GEDII_a/datasets/a03/lc.htm
37	X28 2 Temp Mean 01 deg C	Mean monthly temperature (0.1°C) calculated using data from ftp://ftp.ngdc.noaa.gov/Solid_Earth/Ecosystems/GEDII_a/datasets/a03/lc.htm
38	X30 1 AET Mean mm	Mean monthly AET (Actual Evapotranspiration Rate) from 1920 to 1980 (mm) calculated using the Global Resource Information Database of UNEP and is available from http://www.grid.unep.ch/data/grid/gnv183.php)
39	X30 2 PET Mean mm	Mean monthly PET (Potential Evapotranspiration Rate) from 1920 to 1980 (mm) calculated using the Global Resource Information Database of UNEP and is available from http://www.grid.unep.ch/data/grid/gnv183.php)
