# Supplementary material for: The contribution of historical processes to contemporary extinction risk in placental mammals
Source: Science. Author manuscript; Available in PMC 2023 May 15. (PMC10184782; doi:10.1126/science.abn5856)
Supplement: tables S5 and S6 [file NIHMS1893031-supplement-tables_S5_and_S6.pdf]

Table S5. Machine learning models to predict conservation status from genomic summary statistics, genomic metrics in homologous 50KB windows, and ecological variables across mammal species. Performance was measured by the median AUROC across five training-test replicates. The first AUROC value is for models where missing data was imputed by the median of the lowest taxonomic level, the second value in parentheses is for models with missing data imputed across all species. Data types included in the models were grouped as window-based metrics (Genomic windows), 13 genome-wide summary statistics (Genomic summary), and 39 ecological variables (Ecological).

			**Data types**		
**Model**	**AUROC**	**N**	**Ecological**	**Genomic summary**	**Genomic windows**
Ecological variables	0.88 (0.88)	212	x		
Genomic summary statistics + ecological variables	0.86 (0.86)	210	x	x	
Windows-based mean phyloP + genomic summary statistics	0.77 (0.8)	236		x	x
Windows-based mean phyloP	0.78 (0.77)	236			x
Windows-based mean phyloP + ecological variables	0.73 (0.75)	208	x		x
Windows-based missense substitutions + ecological variables	0.78 (0.74)	208	x		x
All five genomic windows-based metrics + genomic summary statistics	0.73 (0.74)	195		x	x
Windows-based missense substitutions + genomic summary statistics + ecological variables	0.78 (0.74)	208	x	x	x
All five genomic windows-based metrics	0.74 (0.73)	195			x
Windows-based missense substitutions	0.69 (0.73)	236			x
Three best genomic windows-based metrics + ecological variables	0.82 (0.73)	173	x		x
Windows-based mean phyloP + genomic summary statistics + ecological variables	0.75 (0.73)	208	x	x	x
Windows-based conserved missense substitutions + ecological variables	0.82 (0.72)	208	x		x

Model	AUROC	N	Data types		
			Ecological	Genomic summary	Genomic windows
Three best genomic windows-based metrics + genomic summary statistics + ecological variables	0.78 (0.72)	173	x	x	x
All five genomic windows-based metrics + ecological variables	0.79 (0.71)	172	x		x
All five genomic windows-based metrics + genomic summary statistics + ecological variables	0.77 (0.71)	172	x	x	x
Three best genomic windows-based metrics	0.82 (0.7)	196			x
Windows-based RoH + ecological variables	0.81 (0.7)	174	x		x
Windows-based conserved missense substitutions + genomic summary statistics	0.76 (0.7)	236		x	x
Three best genomic windows-based metrics + genomic summary statistics	0.79 (0.69)	196		x	x
Windows-based missense substitutions + genomic summary statistics	0.72 (0.69)	236		x	x
Windows-based conserved missense substitutions + genomic summary statistics + ecological variables	0.81 (0.69)	208	x	x	x
Windows-based conserved missense substitutions	0.7 (0.67)	236			x
Windows-based RoH + genomic summary statistics + ecological variables	0.83 (0.66)	174	x	x	x
Genomic summary statistics	0.68 (0.65)	236		x	
Windows-based RoH + genomic summary statistics	0.78 (0.63)	197		x	x
Windows-based heterozygosity + ecological variables	0.68 (0.61)	174	x		x
Windows-based heterozygosity + genomic summary statistics + ecological variables	0.7 (0.61)	174	x	x	x

			Data types		
**Model**	**AUROC**	**N**	**Ecological**	**Genomic summary**	**Genomic windows**
Windows-based RoH	0.78 (0.59)	197			x
Windows-based heterozygosity + genomic summary statistics	0.78 (0.53)	197		x	x
Windows-based heterozygosity	0.79 (0.52)	197			x

Table S6. Performance (median AUROC across five training-test replicates) of machine learning models to predict conservation status across the same set of species, comparing models with genomic variables only, ecological variables only, and combining both ecological and genomic variables.

Genomic variable type	Genomic only	Ecological only	Ecological + Genomic	# species
Genome-wide summary statistics	0.71	0.83	0.85	210
Window-based Missense substitutions	0.75	0.85	0.74	209
Window-based Mean phyloP of substitutions	0.73	0.82	0.78	209
Three best window-based genomic metrics	0.70	0.83	0.73	174
All five window-based genomic metrics	0.69	0.80	0.68	173
Window-based RoH	0.67	0.82	0.72	175
Window-based Heterozygosity	0.64	0.80	0.75	175
Window-based conserved missense substitutions	0.64	0.84	0.75	209
